# Mutation-Attention (MuAt): deep representation learning of somatic mutations for tumour typing and subtyping

**DOI:** 10.1186/s13073-023-01204-4

**Published:** 2023-07-07

**Authors:** Prima Sanjaya, Katri Maljanen, Riku Katainen, Sebastian M. Waszak, Genomics England Research Consortium, J. C. Ambrose, P. Arumugam, R. Bevers, M. Bleda, F. Boardman-Pretty, C. R. Boustred, H. Brittain, M. A. Brown, M. J. Caulfield, G. C. Chan, A. Giess, J. N. Griffin, A. Hamblin, S. Henderson, T. J. P. Hubbard, R. Jackson, L. J. Jones, D. Kasperaviciute, M. Kayikci, A. Kousathanas, L. Lahnstein, A. Lakey, S. E. A. Leigh, I. U. S. Leong, F. J. Leong, F. Maleady-Crowe, M. McEntagart, F. Minneci, J. Mitchell, L. Moutsianas, M. Mueller, N. Murugaesu, A. C. Need, P. O’Donovan, C. A. Odhams, C. Patch, D. Perez-Gil, M. B. Perez-Gil, J. Pullinger, T. Rahim, A. Rendon, T. Rogers, K. Savage, K. Sawant, R. H. Scott, A. Siddiq, A. Siddiq, S. C. Smith, A. Sosinsky, A. Stuckey, M. Tanguy, A. L. Taylor Tavares, E. R. A. Thomas, S. R. Thompson, A. Tucci, M. J. Welland, E. Williams, K. Witkowska, S. M. Wood, M. Zarowiecki, Lauri A. Aaltonen, Oliver Stegle, Jan O. Korbel, Esa Pitkänen

**Affiliations:** 1 Institute for Molecular Medicine Finland (FIMM), HiLIFE, University of Helsinki, Helsinki, Finland; 2 Applied Tumor Genomics Research Program, Faculty of Medicine, University of Helsinki, Helsinki, Finland; 3 iCAN Digital Precision Cancer Medicine Flagship, Helsinki, Finland; 4 Department of Medical and Clinical Genetics, Faculty of Medicine, University of Helsinki, Helsinki, Finland; 5 Centre for Molecular Medicine Norway (NCMM), Nordic EMBL Partnership, University of Oslo and Oslo University Hospital, Oslo, Norway; 6 Swiss Institute for Experimental Cancer Research School of Life Sciences, École Polytechnique Fédérale de Lausanne (EPFL), Lausanne, Switzerland; 7 Department of Neurology, University of California, San Francisco (UCSF), San Francisco, CA USA; 8 Division of Computational Genomics and Systems Genetics, German Cancer Research Center (DKFZ), Heidelberg, Germany; 9 Genome Biology Unit, European Molecular Biology Laboratory, Heidelberg, Germany; 10 European Molecular Biology Laboratory, European Bioinformatics Institute, Wellcome Genome Campus, Hinxton, Cambridge, UK

**Keywords:** Cancer genomics, Precision cancer medicine, Machine learning, Deep learning, Tumour type prediction, Molecular tumour subtyping, Somatic mutations, Deep neural networks, Whole genome sequencing, Whole exome sequencing

## Abstract

**Background:**

Cancer genome sequencing enables accurate classification of tumours and tumour subtypes. However, prediction performance is still limited using exome-only sequencing and for tumour types with low somatic mutation burden such as many paediatric tumours. Moreover, the ability to leverage deep representation learning in discovery of tumour entities remains unknown.

**Methods:**

We introduce here Mutation-Attention (MuAt), a deep neural network to learn representations of simple and complex somatic alterations for prediction of tumour types and subtypes. In contrast to many previous methods, MuAt utilizes the attention mechanism on individual mutations instead of aggregated mutation counts.

**Results:**

We trained MuAt models on 2587 whole cancer genomes (24 tumour types) from the Pan-Cancer Analysis of Whole Genomes (PCAWG) and 7352 cancer exomes (20 types) from the Cancer Genome Atlas (TCGA). MuAt achieved prediction accuracy of 89% for whole genomes and 64% for whole exomes, and a top-5 accuracy of 97% and 90%, respectively. MuAt models were found to be well-calibrated and perform well in three independent whole cancer genome cohorts with 10,361 tumours in total. We show MuAt to be able to learn clinically and biologically relevant tumour entities including acral melanoma, SHH-activated medulloblastoma, *SPOP*-associated prostate cancer, microsatellite instability, POLE proofreading deficiency, and *MUTYH*-associated pancreatic endocrine tumours without these tumour subtypes and subgroups being provided as training labels. Finally, scrunity of MuAt attention matrices revealed both ubiquitous and tumour-type specific patterns of simple and complex somatic mutations.

**Conclusions:**

Integrated representations of somatic alterations learnt by MuAt were able to accurately identify histological tumour types and identify tumour entities, with potential to impact precision cancer medicine.

**Supplementary Information:**

The online version contains supplementary material available at 10.1186/s13073-023-01204-4.

## Background

Accurate identification of tumour histological type and molecular subtype is crucial to determining cancer diagnosis, prognosis and treatment choice [1, 2]. In paediatric brain tumours, long-term survival can range from 90% for WNT-medulloblastomas to 40% for Group 3-medulloblastomas [3]. Solid tumours exhibiting microsatellite instability (MSI) resulting from defective mismatch repair (MMR) are susceptible to treatment with PD-1 immune checkpoint inhibitors leading to improved response and survival rates [4, 5]. Moreover, approximately 3–5% of metastatic cancers do not have a clear primary site of origin despite comprehensive clinical workup [6, 7]. These cases, termed cancers of unknown primary (CUPs), present a challenge as targeted treatment options depend on the tissue of origin. CUPs are thus often treated with broad spectrum antineoplastic drugs with limited success, instead of site-specific treatments. Liquid biopsies can be used to detect circulating tumour DNA (ctDNA) originating from cancer cells before metastatic spread and to predict disease outcome [8–10]. Similarly to CUPs, determining the tissue of origin of ctDNA is a key obstacle in enabling clinical action.

Somatic mutations in a cancer cell are the consequence of the mutational processes which acted on its ancestors in the somatic cell tree [11]. Many such processes have been identified, including exogenous processes such as ultraviolet radiation and polycyclic aromatic hydrocarbons in tobacco smoke, and endogenous processes such as spontaneous deamination of methylated cytosines, defective DNA repair, and DNA replication infidelity [12]. These processes can have distinct characteristics in terms of DNA substrate preference (e.g. CpG, mononucleotide microsatellite), mutation type (e.g. single-base substitution, insertion or deletion, or structural alteration) and genomic position (e.g. intronic or late replicating region preference), among others [13]. Typically only a handful of mutational processes are active in a cell of specific type and location within the body and tissue [14, 15]. For instance, skin cells exposed to the sun are susceptible to DNA damage due to ultraviolet radiation, whereas B cells undergo somatic hypermutation affecting predominantly the immunoglobulin heavy chain variable region of the genome. Somatic mutations can thus be informative of the tissues and conditions where the mutations occurred, and consequently, cancer genome sequencing can be used to scrutinize the somatic mutations of a cancer with the prospect of revealing its tissue of origin and molecular subtype.

Recently, several computational methods have been developed to predict tumour types by analysing somatic driver and passenger mutation patterns in next-generation sequencing data [16, 17]. TumorTracer is a random forest classifier combining copy number profiles and nucleotide substitution spectra attaining 85% and 69% accuracy across 6 and 10 primary sites, respectively [18]. Soh et al. predicted tumour types with a support vector machine using information on somatically mutated genes resulting in 49% accuracy in 28 tumour types, with addition of copy number profiles increasing accuracy to 78% [19]. Especially in whole cancer genomes, the mutational landscape is dominated by passenger mutations which are highly informative of the tissue-of-origin. As part of the Pan-Cancer Analysis of Whole Genomes project (PCAWG), Jiao et al. explored tumour type prediction in 2606 tumours representing 24 tumour types and achieved 88% accuracy in an independent set of tumour whole genomes with a deep neural network model which takes as input counts of mutation types and their binned genomic positions in each tumour [20]. Tumours exhibiting MSI were removed from data prior to model training. Both Jiao et al. and Salvadores et al. [21] found the utility of driver mutations in accurately predicting tumour types to be limited due to the relatively small number of driver alterations per tumour, few recurrent driver alterations, and lack of strong tumour type specificity for cancer driver genes. Recently, Danyi et al. showed data augmentation to be an effective strategy for tumour typing with sparse sequencing data such as sequencing of ctDNA [22].

While supervised approaches have been developed to predict tumour subtypes [23–25], unsupervised methods are more common due to lack of labelled subtype data. In unsupervised tumour subtyping, one typically aims to find a compact set of latent factors explaining the observed data, often compassing multiple modalities [26], and then identifying subtypes using latent factors. Recent subtyping methods have employed matrix factorization [27], clustering [28], deep autoencoders [24], and adversarial learning [29] of multiomics data. Discovery of prognostic subtypes has been done by weighting or selecting features based on survival [24, 30]. Sequence context of somatic mutations has been shown to be informative in subtyping of breast cancers [31].

Here, we developed a novel deep neural network (DNN) model, termed Mutation-Attention (MuAt), which allows us to predict tumour types from cancer whole-genome and whole-exome sequencing data. It leverages the ability of DNNs to learn in a supervised setting representations that can be used to explore and explain the structure of input data beyond class labels. MuAt utilizes the attention mechanism [32, 33], which allows the model to focus on data elements which are important to solving the learning task at hand, and can lead to improved model performance and explainability [34]. MuAt is able to integrate single-nucleotide and multi-nucleotide substitutions (SNVs/MNVs), short insertions and deletions (indels), structural variant (SV) breakpoints, and combinations of these primary genetic alterations by learning multimodal data embeddings [35–37]. These embeddings integrate mutation type and genomic position information at a per-mutation level, instead of the more common approach of representing types and positions as aggregated counts [20, 21, 35, 36]. A recent method utilized multiple instance learning of mutation embeddings to predict tumour-level attributes including the presence of driver mutations, tumour purity and clonality, and MSI status from a set of genomic variants given their types and positions [37].

In this work, we demonstrate the utility of a supervised approach to compute tumour-level representations from mutation-level representations with the attention mechanism for precision cancer medicine tasks. These tasks include tumour type identification and distinguishing diverse set of tumour characteristics such as molecular subtypes and DNA repair deficiencies without specifically training for each individual characteristic.

Our models achieve high accuracy in predicting tumour types, with top-1 and top-3 accuracies of 88.8% and 96.1% in the 24 tumour types that were studied within the PCAWG consortium. MuAt further outperforms the previous state-of-the-art approaches for cancer types that have been challenging to predict such as tumours with MSI. We investigate the utility of our models in tumour exome sequencing data from the TCGA consortium, achieving 64.1% accuracy across 20 tumour types. Exploring the representations learnt by MuAt, we show that the model learns to differentiate tumour subtypes which were not given as input information. These subtypes include tumours driven by somatic and germline mutations such prostate cancers with somatic *SPOP* mutations and pancreatic endocrine tumours with germline *MUTYH* mutations, hypermutable subtypes such as microsatellite-unstable cancers and polymerase $\epsilon$ proofreading deficient tumours, as well as *CCND1*-amplified acral melanomas, and Sonic Hedgehog (SHH)-activated medulloblastomas.

The use of attention mechanism together with the ability to learn representations for different data modalities such as mutation types and positions allows MuAt to represent each mutation as a combination of these modalities. To gain insight into model results, we show that the trained model learns to focus its attention to mutations that are characteristic for each tumour type. MuAt models trained with cancer genomes from PCAWG and TCGA consortiums, an interactive browser, and the source code are available under a permissible licence at GitHub (https://github.com/primasanjaya/mutation-attention).

## Methods

### Data

To train MuAt models, we utilized WGS data from the Pan-Cancer Analysis of Whole Genome (PCAWG) project and WES data from the Pan-Cancer Atlas project of TCGA. PCAWG analysed whole genomes of 2658 human tumours and matched normal samples across 38 tumour types obtained from International Cancer Genome Consortium (ICGC) and The Cancer Genome Atlas (TCGA) donors [38]. The project released a dataset of somatic mutations called uniformly in these tumours containing somatic SNVs, MNVs, indels (<50 bp), SVs and mobile element insertions (MEIs). To train MuAt models, we utilized only tumour types with more than 20 tumours in PCAWG resulting in 2587 tumours across 24 tumour types and 18 primary sites. These tumours harboured a total of 47,646,239 somatic mutations, divided into 41,969,899 SNVs, 826,093 MNVs, 3,720,396 indels, 1,106,598 SVs and 16,735 MEIs, constituting the PCAWG training dataset. We also trained a random forest model to predict tumour types on mutational signatures identified in PCAWG tumours [15]. We used the single-base substitution (SBS), doublet-base substitution (DBS) and small insertion-and-deletion (ID) signatures and limited the data to the same 24 cancer types that were used to train MuAt.

The Pan-Cancer Atlas project of TCGA [39] released the MC3 somatic mutation dataset consisting of a total of 5,717,732 somatic mutations from 8942 tumours across 32 tumour types [40]. We selected the 20 tumour types with more than 100 tumours into our TCGA training dataset, resulting in 7352 tumours and 2,682,344 somatic mutations (2,498,826 SNVs, 46,589 MNVs, and 136,929 indels). No SVs in the data were included in the training dataset due to these events often occurring in the intergenic regions and thus not adequately captured by exome sequencing.

To validate our results with data which was not used in training, we used the whole genomes available in ICGC that were not included in the PCAWG dataset above. Out of 16 tumour types, 5 tumour types were matched to PCAWG tumour types. There were 309 tumours, with a total of 2,806,722 mutations (2,679,477 SNVs, 66,231 MNVs and 43,014 indels). SVs were not available in this dataset.

Furthermore, we evaluated our models on cancer whole genome sequences available through Genomics England (GEL) (release v16, October 13, 2022) [41]. This dataset consists of 17,234 whole-genome sequences of cancers across 23 cancer types. We selected the seven tumour types which matched the types available in PCAWG, resulting in 9796 tumours, with the total of 334,592,186 SNVs available for benchmarking. Somatic variant positions in the GEL dataset were given in GRCh38 reference genome.

Finally, we carried out validation in a cohort of colorectal cancer (CRC) whole genomes, which not used to train the models. This cohort consisted of 256 cancers with somatic variants called with MuTect v1.1.4 (GRCh37) [42]. Somatic variant calls are available in EGA (accession number EGAS00001003010). Further details on the datasets can be found in Additional file 1: Table S1.

### Model

**Fig. 1:**
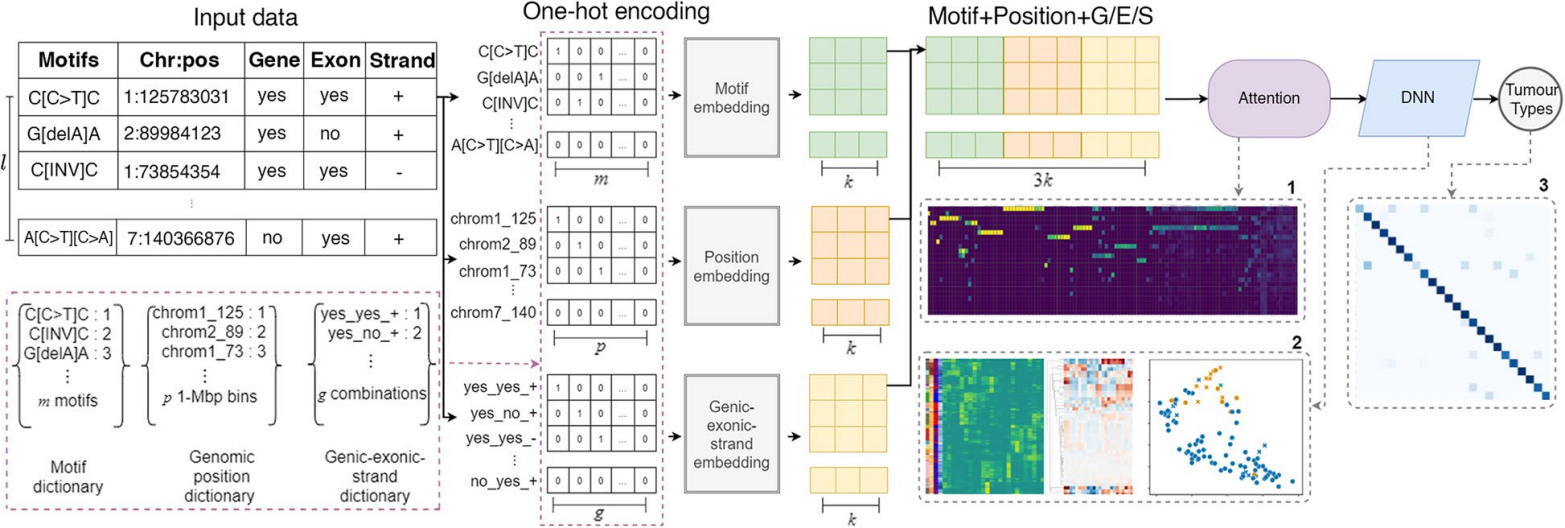
Illustration of the MuAt deep neural network to predict the type of a tumour from its catalogue of somatic mutations. First, mutation data is one-hot encoded. MuAt integrates three data modalities: 3-bp sequence motif, genomic position and genomic annotations. Then, embedded mutation vectors are fed to the attention mechanism. Finally, mutation-level features are combined into tumour-level features, and tumour type is predicted. MuAt models can be interrogated by analysing (1) the attention matrix to recover informative mutations for tumours and tumour types, (2) tumour-level features for tumour subtype discovery and (3) prediction performance

MuAt is a DNN model, which predicts tumour types based on a catalogue of somatic alterations that are observed in a single cancer genome (Fig. 1). We describe here briefly the key aspects of MuAt and provide details in Additional file 2: Fig. S1. The model consists of three consecutive modules. In the first module, mutations are encoded and embedded into a feature space. Three sources of information are used to encode each mutation: (1) mutation type embedded in a three-nucleotide sequence context (e.g. Ap[C>T]pG, Tp[delC]pC), (2) genomic position in 1-Mbp bins and (3) annotations describing whether a mutation occurs in a gene or in an exon, and the coding strand orientation. The supported somatic mutation types are SNVs, indels and SV breakpoints. The MuAt encoding allows for combinations of up to three of these simple mutations to be represented in the sequence context, for instance MNVs (e.g. Cp[C>T]p[C>T]) or >1 bp indels (e.g. [insT]p[insT]p[insT]). Sequence contexts, genomic positions and annotations are represented as one-hot encoded vectors. MuAt learns feature embeddings of these three modalities, which are then concatenated and used as input to the second module.

In the second module, an attention mechanism is used to assign more weight (“soft-select”) to pairs of mutations which are informative in predicting the tumour type, and compute input features for the third module. Attention is defined as

1 $$\begin{aligned} \text {Attention}(Q,K,V)=\sigma \left( \frac{QK'}{\sqrt{d}}\right) V \end{aligned}$$

where *Q*, *K*, *V* are called the query, key and value matrices, respectively, and $\sigma$ denotes the softmax [32, 33]. *Q*, *K* and *V* are all $l \times d$ matrices, where *l* is the number of mutations and *d* is the feature dimension; these matrices are obtained as linear transformations of the input. Product $QK'$ can be seen as a similarity matrix, with the softmax being used to soft-select the most relevant mutations (“keys”) for each mutation (“query”). The attention thus maps each input mutation to a *d*-dimensional feature space defined in terms of similarity to other mutations in the same tumour. Unlike in many natural language processing applications which employ attention [33], no order is imposed on the mutations: MuAt treats somatic mutations analogously to a bag-of-words.

The third module combines the mutation features with fully connected layers yielding tumour-level features. These features are used to compute the final output of the model, which are probabilities over the tumour type labels. The three modules constitute a single model, where all parameters are trained end-to-end with backpropagation and stochastic gradient descent. The trained MuAt model can be interrogated by extracting mutation-level features from the attention module and tumour-level features from the last module. We can project the latter onto a two-dimensional space with UMAP [43] for discovery of tumour subtypes.

### Experimental design and reporting

We were interested in (1) evaluating the contribution of different mutation types in prediction performance, (2) finding hyperparameters which result in best prediction performance, (3) comparing MuAt with existing models, (4) how to best interpret the trained MuAt models, and whether the features learnt by MuAt are compatible with previous findings. This section provides details how the experiments to answer these questions were prepared.

### Data preprocessing

#### Preparing MuAt inputs from somatic variant callsets

For each somatic variant call in the datasets, mutational sequences $s \in \Sigma ^3$ are drawn from alphabet $\Sigma =$ {A, C, G, T} $\cup \, \mathcal {M}$. Mutation symbols $\mathcal {M}$ consist of six substitutions described with respect to the pyrimidine base (i.e. C:G>A:T, C:G>G:C, C:G>T:A, T:A>A:T, T:A>C:G and T:A>G:C); deletions of A, C, G and T; insertions of A, C, G, T; breakpoints for four types of structural variants (i.e. deletions, duplications, inversions and translocations); and retrotransposon insertions (i.e. L1, Alu and SINE-VNTR-Alus (SVA)). This encoding allows representing both simple and complex mutations. For instance, the substitution ApCpG>ApTpG would be encoded as A[C>T]G, a diadenine deletion preceded by a cytosine as C[del A][del A], and a deletion breakpoint with a C>G substitution followed by a thymine as [SV_del][C>G]T, where {[C>T], [del A], [SV_del]} $\subset \mathcal {M}$.

In experiments, we also considered non-mutation events as negative examples. These are constructed by randomly selecting positions where there is a mutation in one tumour (e.g. A[C>T]G at chr1:11,235,813), encoding this position without mutational symbols (e.g. ACG at chr1:11,235,813) and placing it into another tumour’s mutational catalogue. When injecting negative examples into a tumour, we add the median number of variants per type in the dataset as negatives. Specifically, if the dataset has a median of 1000 SNVs and 100 indels, then each tumour will receive a total of 1100 negative examples, where 1000 were picked from random SNVs in other tumours, and the remainder from random indels. Genomic positions are represented in 1-Mbp bins. For instance, the position (chr1, 11,235,813) would be encoded as the token “chr1_11”. This token is used to encode all mutations occurring in chr:11,000,000–11,999,999. Since all models were trained on somatic variants with genomic positions in GRCh37 coordinates, we converted positions to GRCh37 coordinates with Python package “liftover” for any data in GRCh38.

We experimented with mutational annotations consisting of indicators whether the mutation occurs in a gene (“genic”) or in an exon (“exonic”). In addition, we categorize each mutation into one of four mutually exclusive classes (“strand”): mutation’s pyrimidine reference base is on the (1) same or (2) opposite strand as a gene, or (3) mutation overlaps two genes on opposite strands, or (4) mutation is intergenic. This annotation attempted to capture transcriptional strand biases associated with some mutational mechanisms [44].

Each of the three input modalities (mutation motif, position, annotations) was one-hot encoded separately using token dictionaries. The dictionary of positions consisted 2915 tokens for all 1-Mb genomic bins. Mutational motif dictionary consisted of 3692 tokens including 96 SNVs, 2170 MNVs, 1160 indels, 233 SVs and 33 MEIs. Finally, the annotation dictionary contained 2$\times$2$\times$4=16 values for the possible combinations of genic, exonic and strand attributes.

#### MuAt hyperparameter search, model training and validation

We performed search for MuAt hyperparameters over embedding dimensions {128, 256, 512}, number of encoder layers {1, 2, 4}, number of attention heads {1, 2}, number of fully connected layers {1, 2} and mutation types to be included in the input. Additional file 2: Fig. S2 shows the mutation type combinations for PCAWG (15 combinations) and TCGA datasets (9 combinations). We set the learning rate to $6 \times 10^{-4}$, momentum to 0.9, and minibatch size to one, training for 150 epochs. Maximum number of mutations MuAt was able to process per tumour in our experiments was 5000, limited by the memory on GPUs available to us (see Programming environment). MuAt parameters were optimized with stochastic gradient descent minimizing cross-entropy loss

2 $$\begin{aligned} L = H(\textbf{y}, \hat{\textbf{y}}) = - \sum _{i} y_{i} \log (\hat{y}_{i}) \end{aligned}$$

where $\hat{y}_i$ is predicted probability for tumour type *i*, and $y_{i} \in \{0, 1\}$ denotes whether *i* is the correct tumour type.

To perform cross-validation, we split the data equally into ten folds in each cancer type and combined the *i*’th folds from each cancer type. The exact same splits were used to train all models (MuAt, DNN, RF). In each combined fold, we trained a model for each combination of the hyperparameter choices listed above, and the model achieving the best accuracy in the validation set was selected from each fold. The hyperparameters and prediction performance for each fold are given in Additional file 1: Table S2.

To create the MuAt and DNN models used to evaluate prediction performance, and to investigate the tumour-level features learnt by MuAt, we created ensemble models from models obtained in cross-validation. We first chose the best models from each fold as the components of the ensemble. Then, ensemble prediction score was computed by summing the class prediction scores (logits) of these component models, with ensemble prediction corresponding to the tumour type receiving the highest ensemble prediction score.

The model selected for analysis of tumour typing performance in sparse data was trained on SNVs, MNVs, indels and SVs/MEIs, genomic positions and mutation annotations using the PCAWG dataset. This model contained one attention head and two encoder layers, embedding dimension 512, resulting in 28,458,520 trainable parameters.

#### Comparing MuAt with other models

We compared MuAt to deep neural network and random forest (RF) models proposed by Jiao et al. [20]. We further evaluated the contribution of attention mechanism to MuAt performance. In [20], a total of 150 SNV features were used (SNV150) containing the six possible single-nucleotide substitutions (C>A, C>G, C>T, T>A, T>C, T>G), SNVs together with either the flanking 5′ or 3′ base (4$\times$6 + 6$\times$4 = 48 features), and SNVs together with both the 5′ and 3′ flanking bases (4$\times$6$\times$4 = 96 features). To evaluate the contribution of the attention encoder layer, we compared a MuAt model with only one encoder layer to a model without any encoder layers.

To investigate whether mutational signatures could be used to predict tumour types in PCAWG, we created a random forest model on single-base substitution (SBS), doublet-base substitution (DBS) and small insertion-and-deletion signatures (ID) extracted in PCAWG tumours [15]. We used each signature type separately and combined with other signature types. We performed 10-fold cross-validation to train all models, using the same fold splits as for other models. DNN model hyperparameters were optimized in each fold as described in [20]. To train RF models, we used the random forest implementation in scikit-learn package with default parameters.

We report the performance in terms of accuracy $TP + TN / (TP + TN + FP + FN)$, precision $TP/(TP+FP)$, recall $TP/(TP+FN)$ and F1 score $2TP/(2TP+FP+FN)$, where *TP*, *TN*, *FP*, *FN* are the number of true positives and negatives, and false positives and negatives, respectively. Top-*k* accuracies were calculated such that the prediction was deemed correct when the correct class is among the *k* highest scoring predictions.

#### Evaluating MuAt in sparse data

To test the performance of MuAt in sparse data, and further to test transfer learning from WGS to WES data, we selected 13 common tumour types existing in both PCAWG and TCGA dataset (Additional file 1: Table S3). With the same hyperparameters setup as mentioned previously, we retrained the model, as well downsampled validation set with fixed number of mutations $n \in$ {10, 50, 100, 300, 1000, 2500, 4000, 5000}, where 5000 is the maximum capacity of MuAt. Results for transfer learning are shown in Additional file 2: Fig. S3. This setup also used for testing the contribution of the attention mechanism to prediction performance (Fig. 3 and Additional file 2: Fig. S4.

#### Analysis of MuAt tumour-level features

To visualize the tumour-level features learnt by the MuAt ensemble model, we extracted the outputs of the layers (*n*=240 values) before the final prediction from all ten ensemble component models (Additional file 2: Fig. S1). This yielded a feature vector of length 240 for each tumour, which we projected onto a two-dimensional space with UMAP [43]. Interactive UMAP visualizations are available at https://github.com/primasanjaya/mutation-attention.

To reduce complexity of the 240-dimensional feature space for further analysis, we utilized principal component analysis and non-negative matrix factorization (NMF). NMF analysis was carried out with Python sklearn library, minimizing the Frobenius norm. A hyperparameter search was carried out over the number of components to extract in PCAWG data, revealing a knee point at approximately 14 components, with 50 components showing reconstruction error convergence. These 50 components, called MuAt factors M1,$\ldots$,M50, were then extracted from the MuAt PCAWG ensemble model.

Association of MuAt factors with mutational signatures was examined by fitting a least-squares linear model for each PCAWG single-base signature exposure *s* as $\log (s+1) \sim \text {M1}, \ldots , \text {M50}$. Associations with FDR<10% and, for each signature, the variance explained by the linear model are shown in Fig. 5b.

#### Inspecting attention matrices

We analysed the attention matrices $QK'$ of each tumour in a single MuAt model trained on PCAWG somatic SNVs, MNVs, indels and SVs, choosing the best model from (arbitrarily) the first cross-validation fold for this purpose. We extracted the 5000$\times$5000 matrices $A = (a_{ij})$ and selected the values $a_{ij}$>$0.9 \times \text {max}(A)$ to reduce the size of data. Rows and columns of *A* correspond to mutations of a tumour. We can thus visualize the matrices with respect to different mutational data modalities; in our experiments, we visualized mutational motifs and genomic positions (Fig. 6). Genomic annotations (i.e. genic, exonic and strand attributes) were not visualized.

## Results

### Evaluation of histological tumour typing performance

**Fig. 2:**
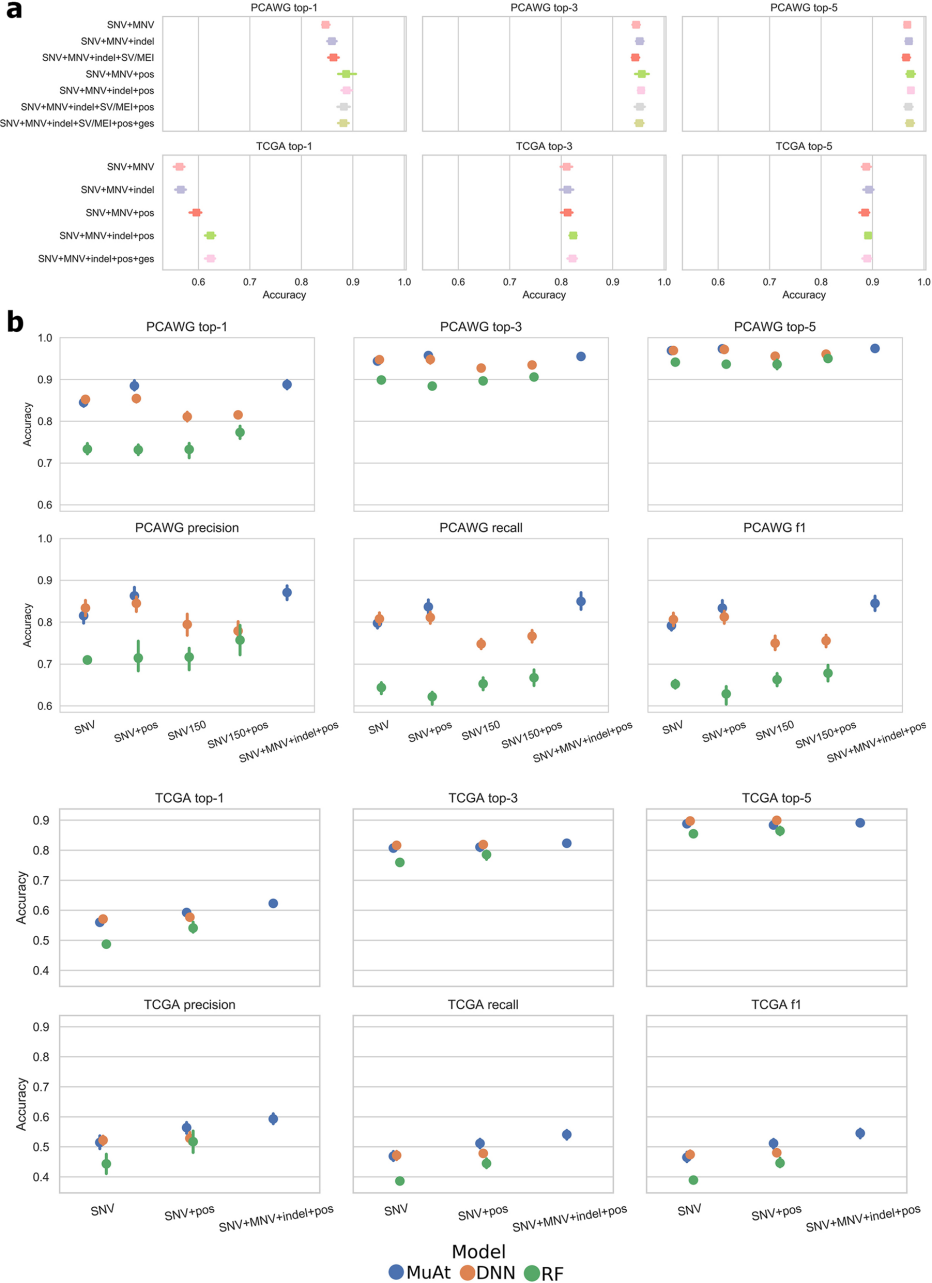
Benchmarking MuAt prediction performance. **a** Top-1, top-3 and top-5 accuracies of MuAt models trained with different mutation types and annotations in PCAWG cancer genomes (top) and TCGA cancer exomes (bottom). Cross-validation accuracies are shown, with the standard deviation of accuracy in cross-validation folds indicated by error bars. **b** Prediction performance of MuAt in PCAWG genomes and TCGA exomes against a recent DNN model [20] and a random forest model (RF). SNV150 indicates the feature set used in [20], “ges” stands for genic, exonic and strand attributes

We first evaluated the contribution of different somatic mutation types and mutation annotations to cross-validated prediction performance. In tumour whole genomes from the PCAWG consortium, the best MuAt performance was obtained with the combination of SNVs, MNVs, indels and genomic position (accuracy 88.8%, 97.4% top-5) (Fig. 2a), although the differences in performance between these scenarios tested were relatively small. In tumour exomes from the TCGA consortium, addition of indels and mutation annotations improved the performance substantially over the other WES models (accuracy 64.1%) (Fig. 2a and Additional file 2: Figs. S2, S5). While predicting the exact tumour type correctly with somatic mutations from exomes compared to whole genomes was more challenging and MuAt did not achieve a high accuracy in predicting the exact tumour type (i.e. top-1 accuracy), the correct tumour type was in the top-5 of MuAt predictions in 90.6% of cases. Furthermore, MuAt predictions were found to be reliable, indicating that the model was well-calibrated (Additional file 2: Fig. S6).

We then benchmarked our method against a recent deep neural network model (DNN, [20]). Here, MuAt outperformed the DNN model, which achieved 85.5% accuracy with PCAWG cancer genomes and 62.8% accuracy in TCGA cancer exomes (Fig. 2b). In [20], the method was evaluated in PCAWG data where MSI tumours had been removed. As the exact type of an MSI tumour can be difficult to predict, and since they represent a clinically important subgroup [45], we kept the MSI tumours in the data. This may explain the difference between our experiment and their reported accuracy of 91% [20] (noted as DNN* in Additional file 1: Table S3a). In top-5 prediction of WGS data, both methods performed similarly (PCAWG MuAt 97.6%, DNN 97.2%), whereas in WES data, DNN was more accurate (TCGA MuAt 90.6%, DNN 91.8%).

We compared the deep neural network-based methods to random forest (RF) models trained on somatic mutations and mutation signatures. The accuracies of RF models on somatic mutations were substantially worse than either MuAt or DNN (75.2% PCAWG, 54.7% TCGA). RF models trained on PCAWG mutational signatures yielded better performance than on mutation counts, but failed to reach accuracies of either MuAt or DNN. RF model trained on combined SBS, DBS and ID signature features achieved accuracy of 81.4%. Individual signature type models had substantially worse accuracies (68.3% SBS, 42.1% DBS, 66.0% ID). Full results are available in Additional file 1: Table S3a.

To gauge the abilities of MuAt models trained with PCAWG data to generalize to unseen data, we evaluated MuAt with whole-genome data not used in training. Here, we evaluated both the performance of models obtained from a cross-validation procedure (“fold models”) and models formed by combining the fold models (“ensemble models,” see the “Methods” section). We first predicted tumour types in the subset of ICGC whole genomes which were not part of PCAWG and where tumour types matched those in the PCAWG (six cohorts, five tumour types). Only models trained with somatic SNVs, or SNVs and MNVs were used, since these were the only mutation types which were available for all six cohorts. In these cohorts, MuAt fold models achieved 85.1% mean accuracy across all tumours, outperforming DNN (82.4%), with the individual cohorts predicted at 74–100% accuracy (Additional file 2: Fig. S7). Ensembling improved accuracies to 90.6% and 86.7% for MuAt and DNN, respectively. We then examined the transferability of MuAt to scenarios where somatic mutation calling workflows differ from those used to generate the PCAWG training data. We tested MuAt, DNN and RF fold and ensemble models on whole genome data of 9796 cancers available in Genomics England, selecting the cancer types matching those in the PCAWG training data (see Additional file 1: Table S1) [41]. MuAt fold models achieved 75.6% mean accuracy compared to 73.0% of DNN and 69.7% of RF (Additional file 1: Table S3c). Again, ensembling improved performance to 81.8% for MuAt, 78.4% for DNN and 72.2% for RF, although tumour type-specific accuracies in GEL remained slightly lower than for the corresponding PCAWG types (e.g. breast cancer 85% in GEL vs 92% in PCAWG, CRC 79% vs 88% and prostate cancer 91% vs 93%) (Figs. S8, S9).

Since raw somatic mutation calls prior to quality filtering were available in GEL, we took the opportunity to evaluate predictive performance also in unfiltered data. We observed the performance of all models trained on PCAWG WGS data to substantially degrade when applied to unfiltered GEL WGS data (Additional file 1: Table S3c). A major cause of this degradation was found to be the presence of false-positive GpTpG>GpGpG somatic mutations in guanine repeats. These variant calls had been identified in GEL as sequencing noise and filtered from the GEL high-quality callset. Removal of these artefacts from the callset partially restored model performance. For instance, MuAt accuracy improved from 47.6% in unfiltered data to 70.6% after artefact removal, compared to the 75.9% accuracy in high-quality data (Additional file 1: Table S3c).

Lastly, to further investigate the potential fragility of deep neural network models to data distribution shifts, we evaluated MuAt and DNN models on a cohort of 256 CRCs not used in training with somatic variants called with MuTect v1.1.4 [42]. MuAt and DNN models reached 76.7% and 75.4% accuracy, respectively (Additional file 1: Table S3b, Fig. S10), similar to performance observed in GEL CRCs (MuAt 78.6%; DNN 68.1%). Model ensembling was found to improve accuracy over single models, resulting in 80.5% for MuAt and 81.6% for DNN (Additional file 1: Table S3b).

**Fig. 3:**
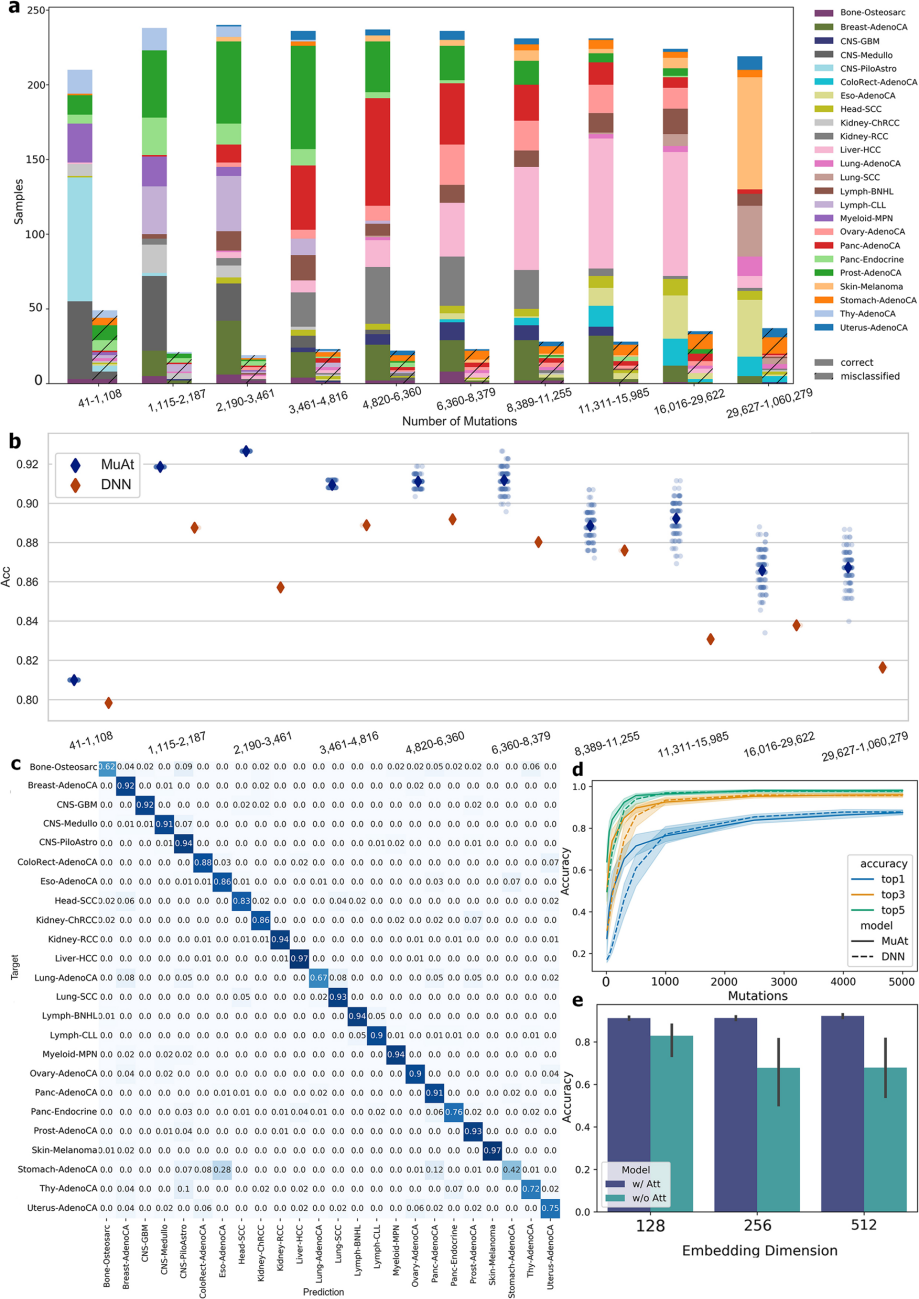
MuAt prediction performance in PCAWG cancer genomes. **a** MuAt predictions stratified by the number of somatic mutations and whether the tumour type was correctly (solid colours) or incorrectly (cross-hatched colours) predicted (top-1). **b** Prediction accuracy (*Y*-axis) of MuAt and DNN by mutational burden (*X*-axis). MuAt results (blue points) show accuracies in repeated independent predictions (*n*=100; diamonds indicate mean accuracies). **c** Confusion matrix of the best-performing MuAt model. **d** Comparison of MuAt and DNN [20] accuracy (*Y*-axis) on sparse data with respect to the number of mutations in downsampled tumours (*X*-axis). Top-1/3/5 accuracies are shown. **e** Accuracy of MuAt with attention (w/ Att) and without (w/o Att), and with respect to the embedding dimensionality

For the remainder of experiments, we proceeded with the MuAt ensemble model trained on SNVs, MNVs, indels and SVs/MEIs, and genomic positions in PCAWG data. We first investigated how mutational burden in each tumour influences prediction performance. As expected, tumours with smallest mutational burden (*n*=259 tumours, <1109 mutations) showed the poorest prediction accuracy with 81% of tumours correctly predicted (Fig. 3a). Many prostate and thyroid cancers and medulloblastomas with low burden were found hard to predict, whereas pilocytic astrocytomas were predicted more accurately.

Similarly, the tumours with the highest burden (*n*=256 tumours, >29,626 mutations) were more difficult to predict (accuracy 85.5%) than the tumours with intermediate burdens (1109–29,626; accuracy 90.4%). This group included many colorectal, stomach and uterine cancers with DNA repair defects leading to hypermutability. Since MuAt capacity is being limited to 5000 mutations that are randomly sampled from the mutation catalogue of the tumour, and this might explain the difficulty in predicting tumours with high mutational burden, we tested the consistency of MuAt predictions by predicting each PCAWG tumour 100 times. Figure 3b shows that although the accuracy varied in tumours with higher mutation burden, MuAt consistently outperformed DNN. In general, the model misclassified similar tumour types, or tumour types exhibiting similar mutational mechanisms, such as lung cancers (lung adenocarcinomas vs squamous cell carcinomas) and gastrointestinal cancers (Fig. 3c).

We investigated the performance of the MuAt PCAWG model in sparse data by subsampling mutations. Figure 3d shows the knee in accuracy (71.1%) to occur already at around maximum number of 500 mutations per sample, and steadily increasing up to 5000 mutations. Notably, top-5 accuracy with 500 mutations was relatively high (95.6%). MuAt performed slightly better than the DNN model in subsampled data. To understand the contribution of the attention mechanism, we evaluated MuAt models with the attention module removed. Such models were found to train and perform poorly compared to full MuAt model (Fig. 3e, Additional file 2: Fig. S4).

### MuAt distinguishes molecular tumour subtypes

**Fig. 4:**
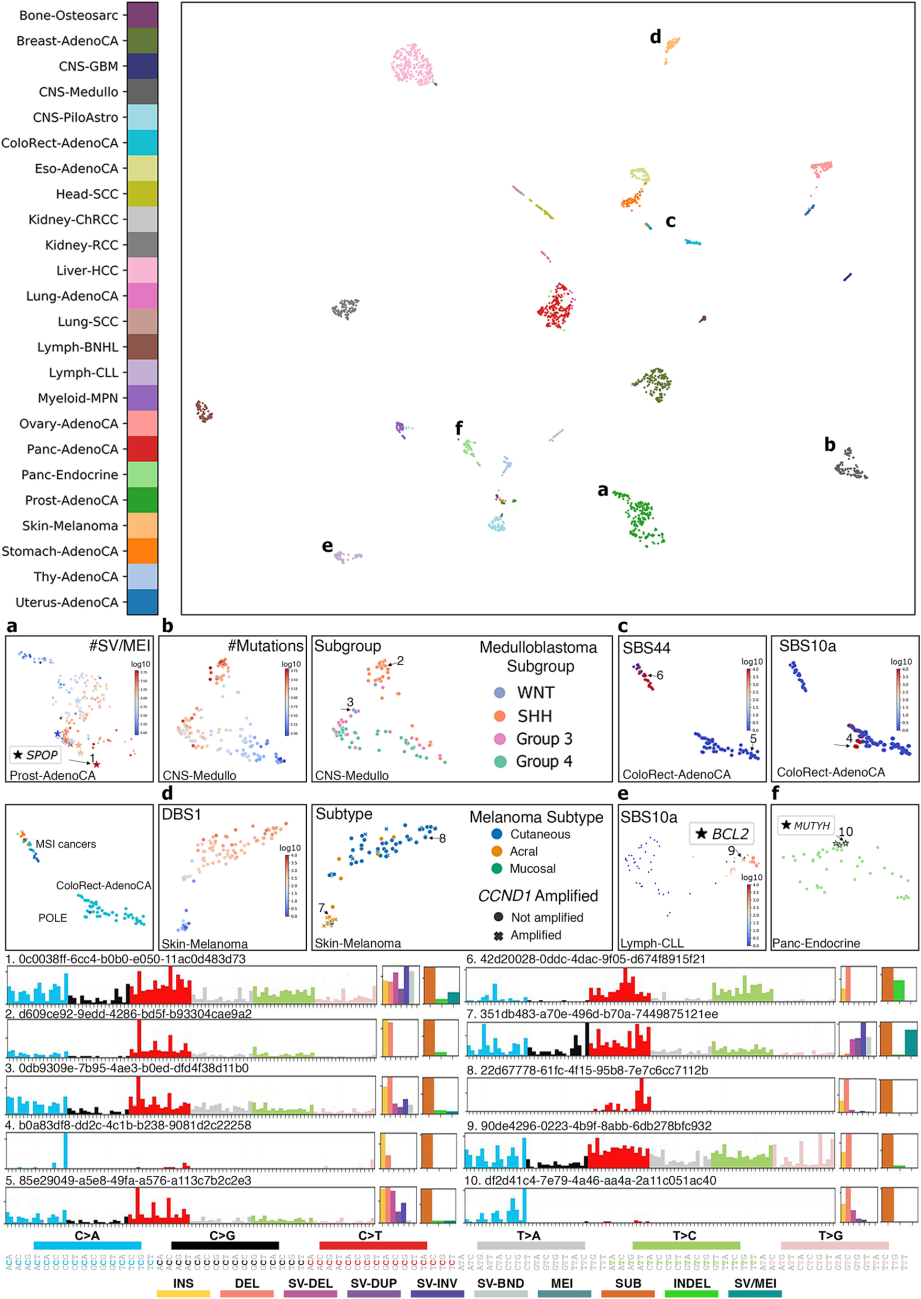
UMAP projection of MuAt tumour-level features in PCAWG data. MuAt recognized tumour subtypes in **a** prostate cancers with a subgroup defined by *SPOP* driver mutations and increased somatic structural variant burden, **b** medulloblastomas, **c** microsatellite-stable colorectal cancers, microsatellite-unstable cancers (MSI) and polymerase $\epsilon$ deficient cancers (POLE), **d** skin melanomas subtypes with *CCND1* amplifications, **e** chronic lymphocytic leukemias associated with somatic hypermutability and **f** pancreatic neuroendocrine tumours with germline *MUTYH* mutations. Specific example tumours are indicated by numbers, with SNV, indel and SV types, and SNV/indel/SV proportions shown. Tumours coloured by a specific single-base or doublet-base signature exposure indicated with SBS or DBS, respectively

We explored the tumour-level MuAt ensemble features (*n*=240) learnt in PCAWG data by projecting the features to a two-dimensional space with UMAP [43]. PCAWG tumours clustered by tumour type as expected due to the high performance of the model in predicting tumour types (Fig. 4). We then investigated whether MuAt features learnt by classifying tumour types could be informative of histological or molecular subtypes even though information on subtypes was not provided during model training. By correlating principal components of MuAt features with known or predicted driver events reported in PCAWG tumours [38] and correcting for tumour histology, we identified a striking association with somatic driver events in *SPOP* (*q*=1.05$\times 10^{-12}$; Additional file 2: Fig. S11, Additional file 1: Table S4), a candidate driver gene in prostate cancer [46]. All twelve prostate cancers with *SPOP* mutations clustered in the MuAt feature UMAP (Fig. 4a, Additional file 2: Fig. S12). These tumours harboured 2.3 times (95% CI, 1.2–4.3x) more somatic SVs than wildtype tumours (Additional file 1: Table S5).

In contrast to tumours with *BRCA1* or *BRCA2* driver events, which harboured 1.6x (95% CI, 1.0–2.4x) and 1.4x (95% CI, 0.9–1.9x) more SVs as well as excess of 10–100 bp deletions (*BRCA1*, 2.2x; *BRCA2*, 7.3x), *SPOP* tumours did not display excess of other somatic mutation types. Instead, *SPOP* tumours constituted a molecular subgroup of prostate cancer characterized by increased somatic structural alteration burden, compatible with previous reports [47]. Prostate cancers with *ERG* driver mutations (*n*=85), mutually exclusive to *SPOP* (OR=0.102, 95% CI, 0.002–0.725, *p*=0.013; [48]), were evenly distributed among the remaining prostate cancers in MuAt clustering.

In medulloblastomas, four subgroups were visible in the feature UMAP (Fig. 4b). One of these was found to correspond to the Sonic Hedgehog (SHH)-activated medulloblastomas of adult patients with mutation landscapes dominated by the age-associated CpG>TpG substitutions [49]. Furthermore, *PTCH1*, *DDX3X* and *SMO* driver mutations characteristic to SHH medulloblastomas, and *PRDM6* enhancer hijacking driver events found in Group 4-medulloblastomas associated with MuAt features (FDR<1%; Additional file 2: Fig. S11). Interestingly, medulloblastomas with *PRDM6* driver alterations (*n*=7) were confined to one of MuAt feature clusters (Additional file 2: Fig. S13). These tumours displayed an increased genome-wide burden of SV duplications (3.7x, 95% CI, 1.6–8.3x), inversions (2.4x, 1.0–5.4x) and translocations (2.4x, 1.0–5.6x), but no excess of other mutation types compared to wildtype medulloblastomas.

MuAt identified tumours with mismatch repair deficiency (MMR) resulting in microsatellite instability as well as tumours exhibiting very high burden of mutations, especially TpCpT>TpApT substitutions characteristic of polymerase $\epsilon$ and $\delta$ proofreading deficient tumours (Fig. 4c). MuAt feature principal components associated with driver mutations in the MMR gene * PMS2* (*q*=1.28$\times 10^{-6}$, Additional file 1: Table S4), as well as with the fraction of microsatellites with somatic mutations (Additional file 1: Table S6), a measure for the level of MSI in a tumour. TpCpT>TpApT substitutions positively associated with MuAt feature principal components at 1% FDR (Additional file 1: Table S7).

In skin melanomas, we observed acral melanomas to cluster by MuAt features (Fig. 4d). Tumours in this subgroup displayed many somatic SVs and amplifications of *CCND1*, a common alteration in acral melanomas associating with ulceration and localized metastasis [50]. The only mucosal melanoma in the data harboured a *CCND1* amplification and clustered with the acral melanomas. The remaining melanomas, mostly of the cutaneous subtype, had a high number of CpC>TpT dinucleotide substitutions compatible with signature DBS1 due to UV light exposure. Finally, in chronic lymphocytic leukemias, MuAt differentiated tumours with patterns of somatic mutations that had occurred in B cells during *IGH* gene rearrangements (Fig. 4e) [51].

In pancreatic neuroendocrine tumours (PanNETs), we discovered four patients with tumours clustering in MuAt feature space to harbour germline mutations in *MUTYH* (p.Tyr176Cys, two patients; p.Pro292Leu; c.924+3A>C) (Fig. 4f, Additional file 2: Fig. S14) (Additional file 1: Table S8). All four tumours showed loss-of-heterozygosity of *MUTYH*. *MUTYH* encodes a DNA glycosylase involved in base excision repair, and germline *MUTYH* mutations have been implicated in a specific G:C>T:A somatic mutation signature found in PanNETs and colorectal cancers [52, 53]. Consistently with these earlier results, we saw an excess of C>A substitutions in NpCpA and NpCpT contexts in *MUTYH* tumours compared to other PanNETs in PCAWG (*t*=9.63, *p*=6.57$\times 10^{-15}$).

Lastly, we projected both PCAWG and GEL tumour-level features onto the same UMAP to examine how well the two cohorts aligned in the MuAt feature space. Most GEL tumours were found to cluster together with PCAWG tumours in tumour type specific clusters, with a small number of tumours with low prediction confidence placed in-between. GEL squamous cell lung cancers (SCC) clustered together with PCAWG SCCs (87% subtyping accuracy, Additional file 2: Fig. S15). In breast cancers, we observed a partial clustering into lobular and ductal carcinomas.

### MuAt features are informative of mutational patterns and correlate with mutational signatures

**Fig. 5:**
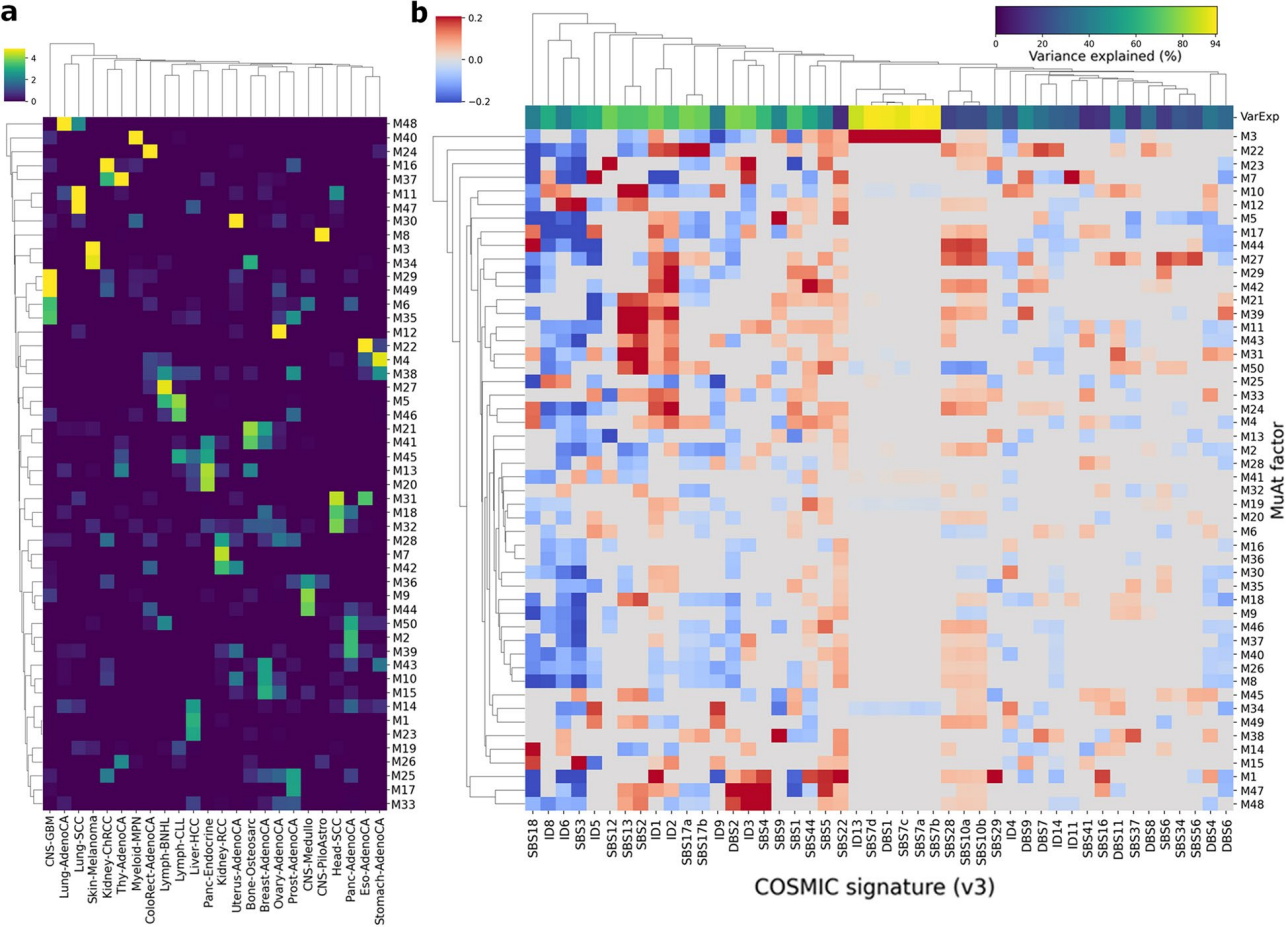
Association of MuAt tumour-level factors with tumour types and mutational signatures. **a** MuAt factor value means (*Y*-axis) by tumour type (*X*-axis) in PCAWG cancer genomes. **b** Coefficients of linear association between MuAt factors and COSMIC SBS mutational signatures (version 2). Top row indicates explained variance in each signature by MuAt factors. Figure shows only signatures with at least one significant association at FDR<10%

MuAt ensemble models are composed of ten MuAt component models and thus output a total of 10$\times$24=240 MuAt tumour-level ensemble features for each input tumour. To understand how these features correlate with mutational patterns and mutational signatures, we first performed non-negative matrix factorization (NMF) on ensemble features extracted from PCAWG tumours. This yielded 50 factors we denote MuAt factors M1,$\ldots$,M50. Many histologies in PCAWG were characterized by high values of specific factors (Fig. 5a, Additional file 2: Fig. S16) such as skin melanomas by factor M3 and M34, lung cancers by M48, pilocytic astrocytomas by M8, gastrointestinal tract cancers by M4, uterine cancers by M30 and liver cancers by M1, M14 and M23.

MuAt factors also associated with specific mutational patterns (Additional file 2: Fig. S17, Additional file 1: Table S9). These included increased burden of structural variants in general (M10, M21, M38), large structural deletions and duplications (M21, M23, M34, M50), and small structural and 10–100 bp deletions (M25). Several factors such as M3 and M42 correlated with higher overall indel burden, whereas factors M8, M13 and M26 correlated with lower indel burden. Specific SNV triplet patterns associating with MuAt factors included TpCpA>TpApA and TpCpT>TpApT (e.g. M44), and TpCpN>TpGpN and TpCpN>TpApN patterns matching the mutational footprint of APOBEC activity (e.g. M11). Many factors represented patterns across different classes of mutations. For instance, factors M3 and M42 strongly associated with increase of both SNVs and indels, whereas M23 positively correlated with SNV and SV burden.

As MuAt factors recapitulated mutational patterns reported in literature, we quantified the independent association of each factor with mutational signatures in PCAWG [14, 15]. Linear models were constructed to explain the number of mutations attributed to each signature in PCAWG tumours (v3; [15]) with MuAt factors. We found many factors to share similarity with single-base (SBS) and doublet-base substitution (DBS), and indel (ID) signatures (Fig. 5; Additional file 2: Fig. S18, Additional file 1: Table S10), with factors explaining over half of variance in 21 signatures (Additional file 2: Fig. S19).

MuAt factor M3 was found to strongly associate with a number of signatures observed in skin melanomas (Fig. 5b). These included single-base (SBS7a-d) and doublet-base substitution (DBS1), and indel (ID3) signatures, all with proposed aetiology of UV light exposure, demonstrating the ability of MuAt to capture different facets of a mutational mechanism in a single factor. Similarly, factors M1, M47 and M48 associated with signatures SBS4, DBS2 and ID3 found in cancers related to tobacco smoking, and factor M12 with homologous recombination DNA repair deficiency related signatures SBS3 and ID6. Other strong associations included APOBEC activity related signatures SBS2 and SBS13 (M11, M10, M31), SBS17a/b possibly related to DNA damage due to reactive oxygen species or 5FU (M22, M4), and SBS12 with currently unknown aetiology present in many liver cancers (M23).

### Attention mechanism captures tumour type specific mutational patterns

**Fig. 6:**
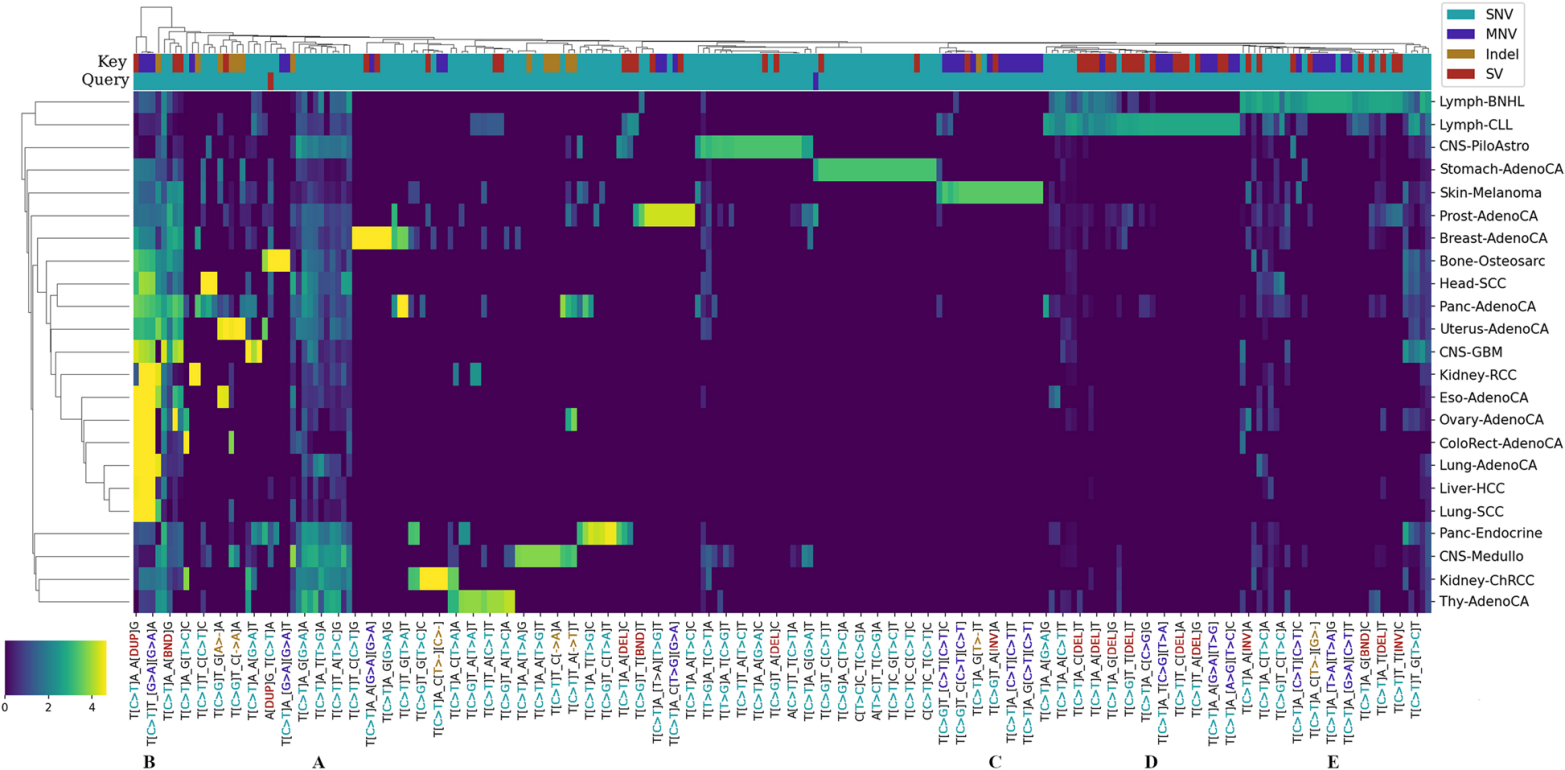
Association between MuAt-derived mutation motifs and tumour types. Attention values for mutation motif pairs (*X*-axis) extracted from the MuAt model trained on PCAWG data. Values have been averaged first over tumours and then over tumour types (*Y*-axis). Types of the key and query mutations (SNV, MNV, indel or SV) are indicated on the two top rows. Every fourth motif pair is labelled as “X_Y” where X and Y are the motifs corresponding to attention query and key, respectively. Label colours indicate mutation types. Motif groups: **A** SNV/SNV and **B** SNV/non-SNV pairs appearing in many tumour types. **C** Motifs with doublet C>T substitutions specific to skin melanomas. **D** and **E** Motifs characteristic to chronic lymphocytic leukemias and Non-Hodgkin lymphomas

To shed light on the mutational patterns learnt by MuAt, we analysed the similarity matrices $QK'$ extracted from the attention module for the tumours in the PCAWG dataset. Figure 6 shows mutation sequence contexts (“motifs”) for mutation pairs which have received most attention stratified by tumour type. The mutation pair motifs most attended to by MuAt contained a SNV paired either with a SNV (52%), MNV (16%), indel (8%), SV (23%) or MEI (0.4%) (Additional file 1: Table S11). All these highly attended MEI events were L1 retrotranspositions occurring in esophageal, pancreatic and prostate adenocarcinomas. Two groups of motifs occurred in most tumour types (Fig. 6 groups A and B). Group A consisted of pairs of SNVs (e.g. (Tp[C>A]pA, Tp[T>G]A)), and group B consisted of SNVs paired with any mutation type (e.g. (Tp[C>A]pA, Ap[BND]pG), where BND denotes a translocation breakend). In addition to these ubiquitous motifs, many tumour types including PanNETs, brain tumours and breast, kidney, prostate and thyroid cancers displayed motifs which were characteristic to each tumour type. Well-known mutational patterns appeared among these motifs, such as the doublet C>T substitutions in skin melanomas (Fig. 6 C). We also investigated occurrence of genomic positions in attention matrices. In chronic lymphocytic leukemias and non-Hodgkin lymphomas, attention focused on mutations occurring in the IGH region (Additional file 2: Fig. S20). These two tumour types displayed both shared and distinct sets of motifs (Fig. 6 D & E). Similarly to motif pair patterns, many tumour types displayed characteristic positional patterns (Additional file 2: Fig. S20).

## Discussion

We developed Mutation-Attention (MuAt), a deep neural network designed to predict tumour types from somatic mutation catalogues while learning informative representations of tumour subtypes. Unlike other recent models, MuAt integrates a variety of heterogeneous information such as mutation type, genomic position and individual mutation annotations rather than representing mutations as aggregated counts. Trained with cancer genomes from the PCAWG consortium, MuAt achieved 89% accuracy in predicting the tumour type in a held-out set of PCAWG tumours (24 types). To evaluate MuAt’s performance with exome sequencing, which is often used in clinical settings [54, 55], we trained and evaluated MuAt with cancer exomes from the TCGA consortium and achieved a top-1 accuracy of 64%. However, top-5 performance in WES data was substantially better, reaching 91% accuracy. The top predictions often included similar tumour types such as lung adenocarcinomas and squamous cell carcinoma, or gastrointestinal tumours. This suggests MuAt results may be informative about tumour origins even if the prediction is not exactly correct.

We benchmarked MuAt against other machine learning (ML) methods utilizing somatic mutations and mutation signatures to predict tumour types. Here, MuAt improved over the previous state-of-the-art in PCAWG, GEL and ICGC tumour genomes, and TCGA tumour exomes. We also observed relatively good performance with downsampled WGS data, which suggests MuAt may be useful for low-coverage WGS data, paediatric tumours, and in cell-free DNA applications where only a fraction of the somatic mutation catalogue of a tumour is captured [22].

Deep learning models have been reported to be fragile, or sensitive to small changes in input data, leading to incorrect or misleading conclusions drawn from model outputs [56, 57]. As model fragility can be a significant challenge when deploying machine learning models for clinical use [58], we investigated whether MuAt models would be able to maintain robustness when faced with shifts in input data distribution. We found that MuAt models trained on WGS data performed well on more than 10,000 independent cancer genomes of Genomics England and ICGC, albeit at a slightly lower level compared to the held-out portion of the training cohort. These differences may be attributed to technical reasons such as the choice of sequencing technology, somatic variant calling methodology and quality control steps, and variability in biological characteristics of the tumour cohorts tested. However, when we tested the methods on somatic variant calls before quality filtering, we discovered that all the tested machine learning models, including MuAt, were sensitive to variant calling artefacts. This finding builds upon previous reports of fragility in ML models and emphasizes the importance of using high-quality data and creating ML models that are robust to input data variability resulting from differences in data generating processes [57].

We showed MuAt tumour-level features to distinguish between tumour subtypes, even if these labels were not available during training. By associating MuAt features with driver events identified in PCAWG, MuAt highlighted prostate cancers driven by *SPOP* mutations [59], characterized by a 2.3-fold increase in somatic SV burden, exceeding the relative burden in tumours with *BRCA1* and *BRCA2* mutations. *SPOP* has a role in DNA damage response [60], and *SPOP* mutated prostate cancers have elevated levels of genomic instability [47, 59]. * SPOP* mutations have been associated with better response to therapies [47, 60], potentially mediated by the increased SV burden.

MuAt stratified medulloblastomas into four clusters, one of which contained seven tumours driven by enhancer hijacking events involving *PRDM6* with a 3.7-fold increase in SV burden. *PRDM6* is activated by enhancer hijacking events in Group 4 medulloblastomas [49]. Whether the frequent structural aberrations in these tumours are due to the *PRDM6* activation, or vice versa, remains to be explored in detail. MuAt also clustered together a group of four pancreatic neuroendocrine tumours harbouring germline mutations of *MUTYH* with loss-of-heterozygosity in the tumours, demonstrating the ability of MuAt to identify tumours with similar germline DNA repair defects [52].

Since MuAt was able to accurately perform tumour typing with somatic mutation catalogues, we hypothesized that the learnt tumour-level features would share a degree of similarity with mutational signatures [14, 15]. We indeed found many MuAt features to associate with COSMIC signatures such as SBS2 and SBS13 (corresponding to APOBEC activity), SBS4 (tobacco smoke) and SBS9 (somatic hypermutation in B cells), although the signatures could not be mapped one-to-one with the features. This is likely due to MuAt not designed to find features which would correspond to independent latent determinants of mutational catalogues similarly to non-negative matrix factorization used in mutational signature methods [14, 15], but instead to predict tumour types as accurately as possible. In contrast to signature analyses requiring a non-trivial refitting step [61, 62], MuAt features for a new sample can be obtained directly given a trained MuAt model. Approaches to learn disentangled representations in deep neural networks may prove fruitful future direction in creating more broadly applicable representations of multiomics data [63, 64].

MuAt’s attention mechanism allowed us to discover aspects of mutation data such as the type and position—or combinations of these—which were informative in predicting each tumour type. We found various types of mutations occurring in the IGH locus to be driving predictions of B cell malignancies. Here, MuAt was able to capture the interaction between specific mutation types and the genomic region characteristic to somatic hypermutation in B cells. MuAt was also able to leverage the rarer mutation types such as L1 retrotranspositions to help identify cancers such as esophageal and other epithelial cancers where these events are relatively common [65–67]. While we were restricted to maximum of 5000 mutations per tumour due to the complexity $O(n^2)$ of the attention mechanism used, recent improvements such as Reformer [68] or Linformer [69] may be used to lift this restriction and to increase MuAt capacity, potentially leading to better performance.

## Conclusion

In conclusion, we have demonstrated how deep representation learning can in large cancer datasets yield features which are useful beyond labelled data, for instance in tumour subtyping. Our method, MuAt, is already able to contribute to multiomics data integration to drive biological discovery and clinical applications by providing informative representations of somatic mutation catalogues of tumours. This can be extended to incorporate additional data on somatic mutations, such as epigenetics, potentially enabling scrutiny of the role of epigenetic interactions in somatic mutagenesis [13, 42, 70–72]. Beyond tumour typing and subtyping, we envision machine learning models such as MuAt to be instrumental in determining cancer prognosis and appropriate treatment choice [73]. As high-throughput patient data accumulates in clinics and cancer projects worldwide, machine learning models able to leverage the massive-scale data will become irreplaceable tools driving digital precision cancer medicine.

## Supplementary Information

**Additional file 1:** **Table S1.** Dataset Information for PCAWG, TCGA, ICGC, and common tumour subtypes in PCAWG and TCGA datasets. **Table S2.** Precision, recall, and f1 scores in the PCAWG and TCGA datasets. **Table S3.** Classification accuracy of the different models benchmarked. **Table S4.** Coefficients with 95% confidence intervals, *p*-values and FDR-adjusted *q*-values of a PCA model associating driver mutation statuses with MuAt features in the PCAWG dataset tumours. **Table S5.** Coefficients with 95% confidence intervals, *p*-values and FDR-adjusted *q*-values of a negative binomial model associating driver mutation statuses with mutation counts in the PCAWG dataset tumours. **Table S6.** Coefficients with 95% confidence intervals, *p*-values and FDR-adjusted *q*-values of a least-squares linear regression model associating MuAt tumour-level features with the fraction of mutated microsatellitesin the PCAWG dataset tumours. **Table S7.** Coefficients with 95% confidence intervals, *p*-values and FDR-adjusted *q*-values of a negative binomial model associating MuAt tumour-level features with mutation counts in the PCAWG dataset tumours. **Table S8.** Pancreatic neuroendocrine tumours in the PCAWG dataset with germline MUTYH driver mutations. **Table S9.** Linear association of MuAt factors with mutation counts. **Table S10.** Linear association of MuAt factors with mutational signatures. **Table S11.** Attention values for mutation type pairs, averaged over tumour types.

**Additional file 2:** **Figure S1.** Architecture of MuAt. **Figure S2.** Top-1, top-3 and top-5 accuracy, precision, recall and F1 scores of MuAt models trained with different mutation types in PCAWG and TCGA data. **Figure S3.** Transfer learning performance. **Figure S4.** MuAt model performance with and without the attention module in PCAWG data with different combinations of mutation types. **Figure S5.** Confusion matrix of MuAt in TCGA data. **Figure S6.** Calibration curve of MuAt trained with PCAWG. **Figure S7.** Confusion matrix for MuAt and DNN in the independent ICGC dataset. **Figure S8.** Confusion matrix for MuAt and DNN trained on PCAWG in GEL. **Figure S9**. Confusion matrix for MuAt trained on PCAWG in GEL. **Figure S10.** Confusion matrix for MuAt and DNN in the Katainen et al dataset. **Figure S11.** Linear association of PCAWG driver events with MuAt tumour-level feature principal components. **Figure S12.** Prostate cancers with SPOP driver events. **Figure S13.** MuAt tumour-level feature UMAP showing medulloblastomas in the PCAWG dataset. **Figure S14.** Pancreatic endocrine tumours of four patients clustering with kidney cancers in MuAt tumour-level feature UMAP. **Figure S15.** MuAt SNV+pos tumour level features on PCAWG and GEL. **Figure S16.** A clustered heatmap of MuAt tumour-level feature in each PCAWG tumours. **Figure S17.** Association of MuAt tumour-level features with mutation counts by type. **Figure S18.** Association of MuAt PCAWG model tumour-level features with COSMIC mutational signatures. **Figure S19.** Variance in the number of mutations attributed to signaturesexplained by MuAt tumour-level features. **Figure S20.** Mean attention values of genomic positions per tumour type in the MuAt PCAWG model.

**Additional file 3.** The Genomics England Research Consortium.

## Data Availability

The data used in this study were downloaded from the ICGC Data Portal (release 28, 27 March 2019) [74], the Genomic Data Commons Data Portal [75], Genomics England (GEL) (release v16, 13 October 2022) [41] and EGA (EGAS00001003010, EGAS00001004710) [76]. MuAt source code, trained models and interactive visualizations are available online [77].

## References

[1] Singh MP, Rai S, Pandey A, Singh NK, Srivastava S (2021). Molecular subtypes of colorectal cancer: An emerging therapeutic opportunity for personalized medicine. Genes Dis..

[2] Jovčevska I (2020). Next generation sequencing and machine learning technologies are painting the epigenetic portrait of glioblastoma. Front Oncol..

[3] Kool M, Korshunov A, Remke M, Jones DTW, Schlanstein M, Northcott PA (2012). Molecular subgroups of medulloblastoma: an international meta-analysis of transcriptome, genetic aberrations, and clinical data of WNT, SHH, Group 3, and Group 4 medulloblastomas. Acta Neuropathol..

[4] Le DT, Uram JN, Wang H, Bartlett BR, Kemberling H, Eyring AD (2015). PD-1 Blockade in Tumors with Mismatch-Repair Deficiency. New England J Med..

[5] Syn NL, Teng MWL, Mok TSK, Soo RA (2017). De-novo and acquired resistance to immune checkpoint targeting. Lancet Oncol..

[6] Greco FA (2013). Molecular diagnosis of the tissue of origin in cancer of unknown primary site: useful in patient management. Curr Treat Options in Oncol..

[7] Priestley P, Baber J, Lolkema MP, Steeghs N, de Bruijn E, Shale C (2019). Pan-cancer whole-genome analyses of metastatic solid tumours. Nature..

[8] Lennon AM, Buchanan AH, Kinde I, Warren A, Honushefsky A, Cohain AT, et al. Feasibility of blood testing combined with PET-CT to screen for cancer and guide intervention. Science. 2020;369(6499). 10.1126/science.abb9601. 10.1126/science.abb9601 PMC7509949 32345712

[9] Bronkhorst AJ, Ungerer V, Holdenrieder S (2019). The emerging role of cell-free DNA as a molecular marker for cancer management. Biomol Detect Quantif..

[10] Meriranta L, Alkodsi A, Pasanen A, Lepistö M, Mapar P, Blaker YN, et al. Molecular features encoded in the ctDNA reveal heterogeneity and predict outcome in high-risk aggressive B-cell lymphoma. Blood. 2021. 10.1182/blood.2021012852. 10.1182/blood.2021012852 34932792

[11] Gerstung M, Jolly C, Leshchiner I, Dentro SC, Gonzalez S, Rosebrock D (2020). The evolutionary history of 2,658 cancers. Nature..

[12] Chatterjee N, Walker GC (2017). Mechanisms of DNA damage, repair, and mutagenesis. Environ Mol Mutagen..

[13] Gonzalez-Perez A, Sabarinathan R, Lopez-Bigas N (2019). Local Determinants of the Mutational Landscape of the Human Genome. Cell..

[14] Alexandrov LB, Nik-Zainal S, Wedge DC, Aparicio SA, Behjati S, Biankin AV (2013). Signatures of mutational processes in human cancer. Nature..

[15] Alexandrov LB, Kim J, Haradhvala NJ, Huang MN, Ng AWT, Wu Y (2020). The repertoire of mutational signatures in human cancer. Nature..

[16] Lee K, Jeong HO, Lee S, Jeong WK (2019). CPEM: Accurate cancer type classification based on somatic alterations using an ensemble of a random forest and a deep neural network. Sci Rep..

[17] Tothill RW, Li J, Mileshkin L, Doig K, Siganakis T, Cowin P (2013). Massively-parallel sequencing assists the diagnosis and guided treatment of cancers of unknown primary. J Pathol..

[18] Marquard AM, Birkbak NJ, Thomas CE, Favero F, Krzystanek M, Lefebvre C (2015). TumorTracer: a method to identify the tissue of origin from the somatic mutations of a tumor specimen. BMC Med Genomics..

[19] Soh KP, Szczurek E, Sakoparnig T, Beerenwinkel N (2017). Predicting cancer type from tumour DNA signatures. Genome Med..

[20] Jiao W, Atwal G, Polak P, Karlic R, Cuppen E, Danyi A (2020). A deep learning system accurately classifies primary and metastatic cancers using passenger mutation patterns. Nat Commun..

[21] Salvadores M, Mas-Ponte D, Supek F (2019). Passenger mutations accurately classify human tumors. PLoS Comput Biol..

[22] Danyi A, Jager M, de Ridder J (2021). Cancer Type Classification in Liquid Biopsies Based on Sparse Mutational Profiles Enabled through Data Augmentation and Integration. Life..

[23] Gao F, Wang W, Tan M, Zhu L, Zhang Y, Fessler E (2019). DeepCC: a novel deep learning-based framework for cancer molecular subtype classification. Oncogenesis..

[24] Ju J, Wismans LV, Mustafa DAM, Reinders MJT, van Eijck CHJ, Stubbs AP (2021). Robust deep learning model for prognostic stratification of pancreatic ductal adenocarcinoma patients. iScience..

[25] Poirion OB, Jing Z, Chaudhary K, Huang S, Garmire LX (2021). DeepProg: an ensemble of deep-learning and machine-learning models for prognosis prediction using multi-omics data. Genome Med..

[26] Argelaguet R, Arnol D, Bredikhin D, Deloro Y, Velten B, Marioni JC (2020). MOFA+: a statistical framework for comprehensive integration of multi-modal single-cell data. Genome Biol..

[27] Le Van T, van Leeuwen M, Carolina Fierro A, De Maeyer D, Van den Eynden J, Verbeke L (2016). Simultaneous discovery of cancer subtypes and subtype features by molecular data integration. Bioinformatics..

[28] Nguyen H, Shrestha S, Draghici S, Nguyen T (2019). PINSPlus: a tool for tumor subtype discovery in integrated genomic data. Bioinformatics..

[29] Yang H, Chen R, Li D, Wang Z. Subtype-GAN: a deep learning approach for integrative cancer subtyping of multi-omics data. Bioinformatics. 2021. 10.1093/bioinformatics/btab109. 10.1093/bioinformatics/btab109 33599254

[30] Arora A, Olshen AB, Seshan VE, Shen R (2020). Pan-cancer identification of clinically relevant genomic subtypes using outcome-weighted integrative clustering. Genome Med..

[31] Zhang Y, Xiao Y, Yang M, Ma J. Cancer mutational signatures representation by large-scale context embedding. Bioinformatics. 2020;36(Supplement_1):i309-i316. 10.1093/bioinformatics/btaa433 PMC7355300 32657413

[32] Bahdanau D, Cho K, Bengio Y. Neural machine translation by jointly learning to align and translate. arXiv:1409.0473. 2014.

[33] Vaswani A, Shazeer N, Parmar N, Uszkoreit J, Jones L, Gomez AN, et al. Attention is all you need. Adv Neural Inf Process Syst. 2017;30:5998–6008.

[34] Payrovnaziri SN, Chen Z, Rengifo-Moreno P, Miller T, Bian J, Chen JH (2020). Explainable artificial intelligence models using real-world electronic health record data: a systematic scoping review. J Am Med Inf Assoc JAMIA..

[35] Kim S, Lee H, Kim K, Kang J. Mut2Vec: distributed representation of cancerous mutations. BMC Medical Genomics. 2018;11(S2). 10.1186/s12920-018-0349-7. 10.1186/s12920-018-0349-7 PMC5918431 29697361

[36] Palazzo M, Beauseroy P, Yankilevich P (2019). A pan-cancer somatic mutation embedding using autoencoders. BMC Bioinformatics..

[37] Anaya J, Sidhom JW, Cummings CA, Baras AS, the AACR Project GENIE Consortium. Aggregation Tool for Genomic Concepts (ATGC): A deep learning framework for sparse genomic measures and its application to tumor mutational burden. 2021. 10.1101/2020.08.05.237206

[38] ICGC/TCGA Pan-Cancer Analysis of Whole Genomes Consortium (2020). Pan-cancer analysis of whole genomes. Nature..

[39] Hoadley KA, Yau C, Hinoue T, Wolf DM, Lazar AJ, Drill E (2018). Cell-of-Origin Patterns Dominate the Molecular Classification of 10,000 Tumors from 33 Types of Cancer. Cell..

[40] Ellrott K, Bailey MH, Saksena G, Covington KR, Kandoth C, Stewart C (2018). Scalable Open Science Approach for Mutation Calling of Tumor Exomes Using Multiple Genomic Pipelines. Cell Syst..

[41] Caulfield M, Davies J, Dennys M, Elbahy L, Fowler T, Hill S, et al. The National Genomics Research and Healthcare Knowledgebase v5, Genomics England. 2020. https://figshare.com/articles/dataset/GenomicEnglandProtocol_pdf/4530893. Accessed 21 Oct 2022.

[42] Katainen R, Dave K, Pitkänen E, Palin K, Kivioja T, Välimäki N (2015). CTCF/cohesin-binding sites are frequently mutated in cancer. Nat Genet..

[43] McInnes L, Healy J, Melville J. UMAP: Uniform manifold approximation and projection for dimension reduction. arXiv:1802.03426. 2018.

[44] Haradhvala NJ, Polak P, Stojanov P, Covington KR, Shinbrot E, Hess JM (2016). Mutational Strand Asymmetries in Cancer Genomes Reveal Mechanisms of DNA Damage and Repair. Cell..

[45] André T, Shiu KK, Kim TW, Jensen BV, Jensen LH, Punt C (2020). Pembrolizumab in Microsatellite-Instability-High Advanced Colorectal Cancer. N Engl J Med..

[46] Wang Z, Song Y, Ye M, Dai X, Zhu X, Wei W (2020). The diverse roles of SPOP in prostate cancer and kidney cancer. Nat Rev Urol..

[47] Boysen G, Barbieri CE, Prandi D, Blattner M, Chae SS, Dahija A, et al. SPOP mutation leads to genomic instability in prostate cancer. eLife. 2015;4. 10.7554/eLife.09207 PMC4621745 26374986

[48] Shoag J, Liu D, Blattner M, Sboner A, Park K, Deonarine L (2018). SPOP mutation drives prostate neoplasia without stabilizing oncogenic transcription factor ERG. J Clin Investig..

[49] Northcott PA, Buchhalter I, Morrissy AS, Hovestadt V, Weischenfeldt J, Ehrenberger T (2017). The whole-genome landscape of medulloblastoma subtypes. Nature..

[50] Vízkeleti L, Ecsedi S, Rákosy Z, Orosz A, Lázár V, Emri G (2012). The role of CCND1 alterations during the progression of cutaneous malignant melanoma. Tumor Biol..

[51] Willis TG, Dyer MJ (2000). The role of immunoglobulin translocations in the pathogenesis of B-cell malignancies. Blood..

[52] Scarpa A, Chang DK, Nones K, Corbo V, Patch AM, Bailey P (2017). Whole-genome landscape of pancreatic neuroendocrine tumours. Nature..

[53] Viel A, Bruselles A, Meccia E, Fornasarig M, Quaia M, Canzonieri V, et al. A Specific Mutational Signature Associated with DNA 8-Oxoguanine Persistence in MUTYH-defective Colorectal Cancer. eBioMedicine. 2017;20:39–49. 10.1016/j.ebiom.2017.04.022 PMC5478212 28551381

[54] Cobain EF, Wu YM, Vats P, Chugh R, Worden F, Smith DC (2021). Assessment of Clinical Benefit of Integrative Genomic Profiling in Advanced Solid Tumors. JAMA Oncol..

[55] Morash M, Mitchell H, Beltran H, Elemento O, Pathak J (2018). The role of next-generation sequencing in precision medicine: a review of outcomes in oncology. J Personalized Med..

[56] DeGrave AJ, Janizek JD, Lee SI (2021). AI for radiographic COVID-19 detection selects shortcuts over signal. Nat Mach Intell..

[57] Pohjonen J, Stürenberg C, Rannikko A, Mirtti T, Pitkänen E (2022). Spectral decoupling for training transferable neural networks in medical imaging. iScience..

[58] Hu Y, Jacob J, Parker GJ, Hawkes DJ, Hurst JR, Stoyanov D (2020). The challenges of deploying artificial intelligence models in a rapidly evolving pandemic. Nat Mach Intell..

[59] Barbieri CE, Baca SC, Lawrence MS, Demichelis F, Blattner M, Theurillat JP (2012). Exome sequencing identifies recurrent SPOP, FOXA1 and MED12 mutations in prostate cancer. Nat Genet..

[60] Clark A, Burleson M (2020). SPOP and cancer: a systematic review. Am J Cancer Res..

[61] Maura F, Degasperi A, Nadeu F, Leongamornlert D, Davies H, Moore L (2019). A practical guide for mutational signature analysis in hematological malignancies. Nat Commun..

[62] Degasperi A, Amarante TD, Czarnecki J, Shooter S, Zou X, Glodzik D (2020). A practical framework and online tool for mutational signature analyses show inter-tissue variation and driver dependencies. Nat Cancer..

[63] Chen RT, Li X, Grosse RB, Duvenaud DK. Isolating sources of disentanglement in variational autoencoders. Adv Neural Inf Process Syst. 2018;31:2615–25.

[64] Van Den Oord A, Vinyals O, et al. Neural discrete representation learning. Adv Neural Inf Process Syst. 2017;30:6306–15.

[65] Doucet-O’Hare TT, Rodić N, Sharma R, Darbari I, Abril G, Choi JA (2015). LINE-1 expression and retrotransposition in Barrett’s esophagus and esophageal carcinoma. Proc Natl Acad Sci..

[66] Rodriguez-Martin B, Alvarez EG, Baez-Ortega A, Zamora J, Supek F, Demeulemeester J (2020). Pan-cancer analysis of whole genomes identifies driver rearrangements promoted by LINE-1 retrotransposition. Nat Genet..

[67] Cajuso T, Sulo P, Tanskanen T, Katainen R, Taira A, Hänninen UA (2019). Retrotransposon insertions can initiate colorectal cancer and are associated with poor survival. Nat Commun..

[68] Kitaev N, Kaiser Ł, Levskaya A. Reformer: The efficient transformer. arXiv:2001.04451. 2020.

[69] Wang S, Li BZ, Khabsa M, Fang H, Ma H. Linformer: Self-attention with linear complexity. arXiv:2006.04768. 2020.

[70] Schuster-Böckler B, Lehner B (2012). Chromatin organization is a major influence on regional mutation rates in human cancer cells. Nature..

[71] Polak P, Karlić R, Koren A, Thurman R, Sandstrom R, Lawrence M (2015). Cell-of-origin chromatin organization shapes the mutational landscape of cancer. Nature..

[72] Guo J, Zhou Y, Xu C, Chen Q, Sztupinszki Z, Börcsök J (2021). Genetic Determinants of Somatic Selection of Mutational Processes in 3,566 Human Cancers. Cancer Res..

[73] Tran KA, Kondrashova O, Bradley A, Williams ED, Pearson JV, Waddell N (2021). Deep learning in cancer diagnosis, prognosis and treatment selection. Genome Med..

[74] The International Cancer Genome Consortium. ICGC Data Portal. 2022. https://dcc.icgc.org/releases/release_28. Accessed 3 Feb 2022.

[75] National Cancer Institute. Genomic Data Commons Data Portal. 2019. https://portal.gdc.cancer.gov/. Accessed 1 Oct 2019.

[76] Tumor Genomics Committee. Somatic variants in 344 colorectal cancer samples. The European Genome-phenome Archive (EGA). 2022. https://ega-archive.org/datasets/EGAD00001006572. Accessed 13 Oct 2022.

[77] Sanjaya P. Mutation-Attention (MuAt). GitHub. 2022. https://github.com/primasanjaya/mutation-attention. Commit: 3f2d561. Accessed 8 Dec 2022

