# Supplementary material for: Mutation-Attention (MuAt): deep representation learning of somatic mutations for tumour typing and subtyping
Source: Genome Med. 2023 Jul 7;15:47. doi: 10.1186/s13073-023-01204-4 (PMC10326961; doi:10.1186/s13073-023-01204-4)
Supplement: Supplementary file 2 — Additional file 2: Figure S1. Architecture of MuAt. Figure S2. Top-1, top-3 and top-5 accuracy, precision, recall and F1 scores of MuAt models trained with different mutation types in PCAWG and TCGA data. Figure S3. Transfer learning performance. Figure S4. MuAt model performance with and without the attention module in PCAWG data with different combinations of mutation types. Figure S5. Confusion matrix of MuAt in TCGA data. Figure S6. Calibration curve of MuAt trained with PCAWG. Figure S7. Confusion matrix for MuAt and DNN in the independent ICGC dataset. Figure S8. Confusion matrix for MuAt and DNN trained on PCAWG in GEL. Figure S9. Confusion matrix for MuAt trained on PCAWG in GEL. Figure S10. Confusion matrix for MuAt and DNN in the Katainen et al dataset. Figure S11. Linear association of PCAWG driver events with MuAt tumour-level feature principal components. Figure S12. Prostate cancers with SPOP driver events. Figure S13. MuAt tumour-level feature UMAP showing medulloblastomas in the PCAWG dataset. Figure S14. Pancreatic endocrine tumours of four patients clustering with kidney cancers in MuAt tumour-level feature UMAP. Figure S15. MuAt SNV+pos tumour level features on PCAWG and GEL. Figure S16. A clustered heatmap of MuAt tumour-level feature in each PCAWG tumours. Figure S17. Association of MuAt tumour-level features with mutation counts by type. Figure S18. Association of MuAt PCAWG model tumour-level features with COSMIC mutational signatures. Figure S19. Variance in the number of mutations attributed to signaturesexplained by MuAt tumour-level features. Figure S20. Mean attention values of genomic positions per tumour type in the MuAt PCAWG model. [file 13073_2023_1204_MOESM2_ESM.pdf]

Supplementary Information

Mutation-Attention (MuAt): deep representation learning of somatic mutations for tumour typing and subtyping

Prima Sanjaya[1,2,3], Katri Maljanen[1,2,3], Riku Katainen[1,2,3,10], Sebastian M. Waszak[4,5,6], Genomics England Consortium[11], Lauri Aaltonen[2,10], Oliver Stegle[7,8], Jan O. Korbel[8,7,9], and Esa Pitkänen[1,2,3,8,*]

[1]Institute for Molecular Medicine Finland (FIMM), HiLIFE, University of Helsinki, Finland

[2]Applied Tumor Genomics Research Program, Faculty of Medicine, University of Helsinki, Finland

[3]iCAN Digital Precision Cancer Medicine Flagship, Finland

[4]Centre for Molecular Medicine Norway (NCMM), Nordic EMBL Partnership, University of Oslo and Oslo University Hospital, Oslo, Norway

[5]Swiss Institute for Experimental Cancer Research, School of Life Sciences, École Polytechnique Fédérale de Lausanne (EPFL), Lausanne, Switzerland

[6]Department of Neurology, University of California, San Francisco, San Francisco, United States

[7]Division of Computational Genomics and Systems Genetics, German Cancer Research Center (DKFZ), Heidelberg, Germany

[8]Genome Biology Unit, European Molecular Biology Laboratory, Heidelberg, Germany

[9]European Molecular Biology Laboratory, European Bioinformatics Institute, Wellcome Genome Campus, Hinxton, Cambridge, UK

[10]Department of Medical and Clinical Genetics, Faculty of Medicine, University of Helsinki, Helsinki, Finland

[*] Corresponding author, E-mail: esa.pitkanen@helsinki.fi. [11] Full list of consortium is available in the Additional File 3

Supplementary Method

MuAt architecture

The architecture of MuAt is shown in Figure 1, and detailed in Figure S1. Input to MuAt is a set of l genomic variants. These variants are described with respect to their mutation type and sequence context (mutation motif), genomic position and mutational annotations. Each modality is one-hot encoded using a respective dictionary, resulting in $l \times m$, $l \times p$, and $l \times g$ one-hot matrices, where m, p and g are the numbers of unique motifs, genomic positions per 1-Mbp bins, and mutation annotations, respectively. These one-hot encoded matrices are then multiplied with embedding matrices ($\{m, p, g\} \times k$), resulting in three $l \times k$ embedding matrices. Embedding matrices are concatenated to obtain an $l \times 3k$ matrix X_E, which is the input to the MuAt attention module.

Query, key and value matrices are computed by multiplying the input embedding matrix X_E with respective $3k \times 3k$ weight matrix, $Q = X_E W_Q$, $K = X_E W_K$ and $V = X_E W_V$. The attention mechanism Eq. 1 is then applied h times, where h is the number of attention heads. The resulting $l \times 3k$ feature matrix is then combined with X_E via skip connections, and fed to batch normalization and fully connected (FC) layers. Finally, the $l \times 3k$ matrix is average-pooled into a $3k$-vector and processed in a fully connected layer to yield f sample-level features (Fig. 4). In our experiments, we used $f = 24$. To obtain the final tumor type predictions, sample-level features are inputted to a fully connected layer, and its outputs are normalized with softmax $\sigma(\mathbf{z})_i = \frac{e^{z_i}}{\sum_j e^{z_j}}$ to obtain probabilities over tumor types.

MuAt model ensembling

To create MuAt ensemble models, we took the best model for each fold found in a 10-fold cross-validation procedure. MuAt ensemble model predictions were obtained by summing tumor type class logit scores, and choosing the tumor type with the maximum value as the predicted type. There are 24*10=240 tumour-level features obtained from a MuAt ensemble model.

Association of MuAt features with driver events and somatic mutation patterns

Driver events identified in PCAWG tumours [38] were associated with principal components of MuAt ensemble model features with least-squares regression. For each pair of MuAt feature principal component (n=240) and driver (n=298), a least-squares linear model $p \sim d + \text{age} + \text{sex} + \text{histology} + g_1 + \ldots + g_{10}$ was fitted, where p is the feature principal component, d is an indicator whether the driver event was detected in the tumour, and g_i is the ith principal

component of patient genotypes computed in PCAWG [38]. Association was computed only for drivers with at least three tumours harboring the driver, resulting in 7,128 models. P-values from all tests were adjusted for multiple testing with Benjamini-Hochberg method. Figure S11 shows the driver coefficients for all feature principal components from all models, as well as a histogram and a quantile-quantile plot of unadjusted p-values against a uniform distribution showing relatively small degree of inflation.

To analyse correspondence of MuAt features with COSMIC signatures, we carried out least-squares linear regression for each signature separately to predict the log-transformed signature value s based on MuAt factors, *i.e.*, $\log(s+1) \sim$ M1 $+\ldots+$ M50. We corrected p-values with the Benjamini-Hochberg method and reported results with false discovery rate (FDR) $<$10% (Figure S18). For each signature, the variance explained by MuAt factors as adjusted R^2 value is given. This analysis was performed for both COSMIC version 2 and version 3 signatures.

Association of MuAt features with mutation counts stratified by type was quantified with negative binomial model to predict mutation count based on MuAt factors M1 $+\ldots+$ M50, showing results with FDR$<$5% in Figure S17. Association with MSI levels was performed by predicting the fraction of mutated microsatellites computed previously [38] from MuAt factors, *i.e.*, MSI $\sim$ M1, …, M50 with a least-squares regression model.

Programming environment

We implemented MuAt with PyTorch 1.8.0 deep learning framework in Python 3.7. To evaluate the model of Jiao *et al.* [20], we used the code provided at `https://github.com/ICGC-TCGA-PanCancer/TumorType-WGS`, and ran it with TensorFlow 2.0 in Python 3.6. For the random forest model, we used scikit-learn 0.21.3. Statistical modelling was done with scipy 1.5.3 and statsmodels 0.12.1 packages. Packages used for data analysis and visualization included pandas 1.3.4, seaborn 0.11.2 and umap-learn 0.5.1. All deep neural network models were trained on NVidia Tesla V100 GPUs with 16 GB memory.

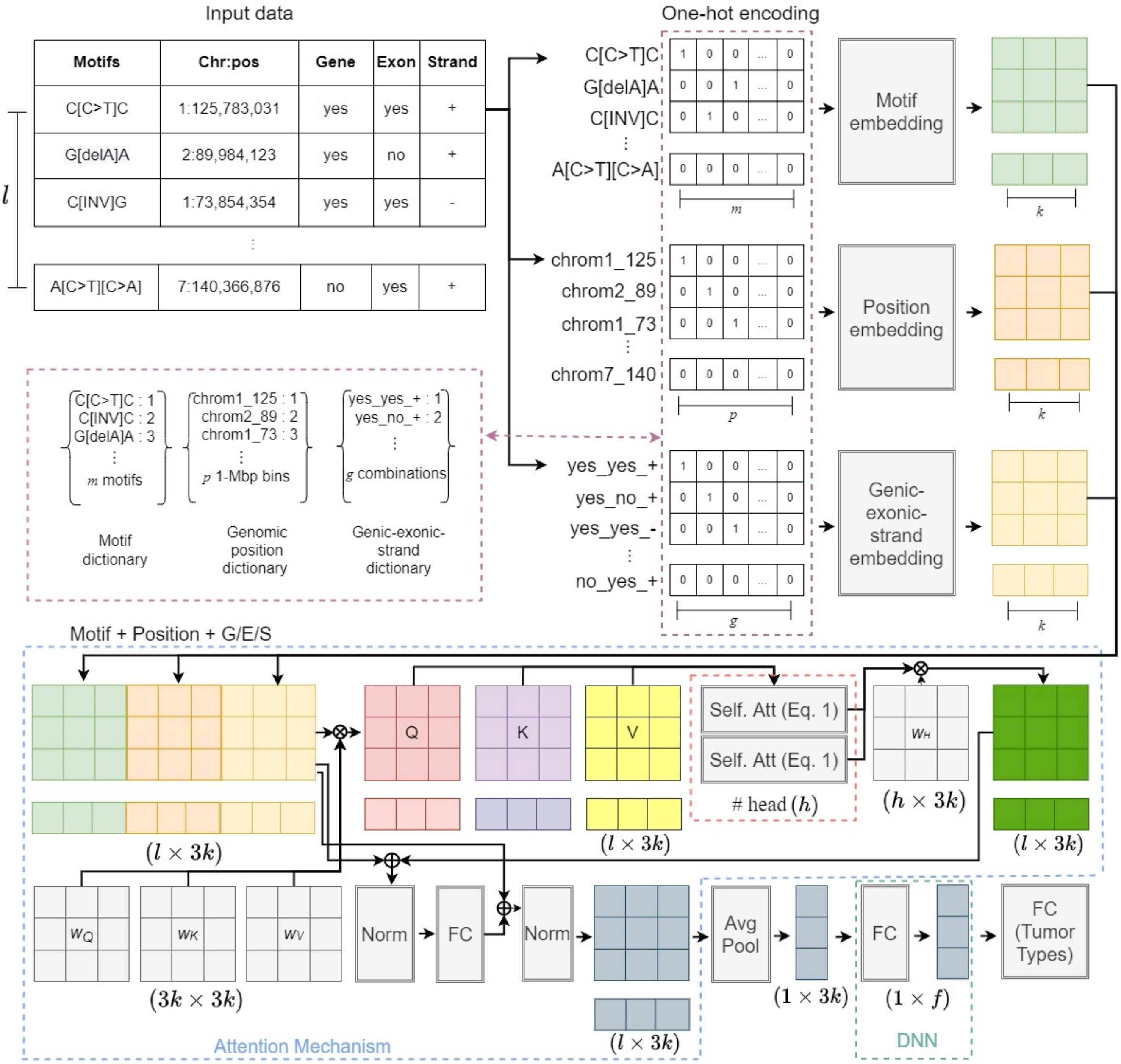

Figure S 1: Architecture of MuAt.

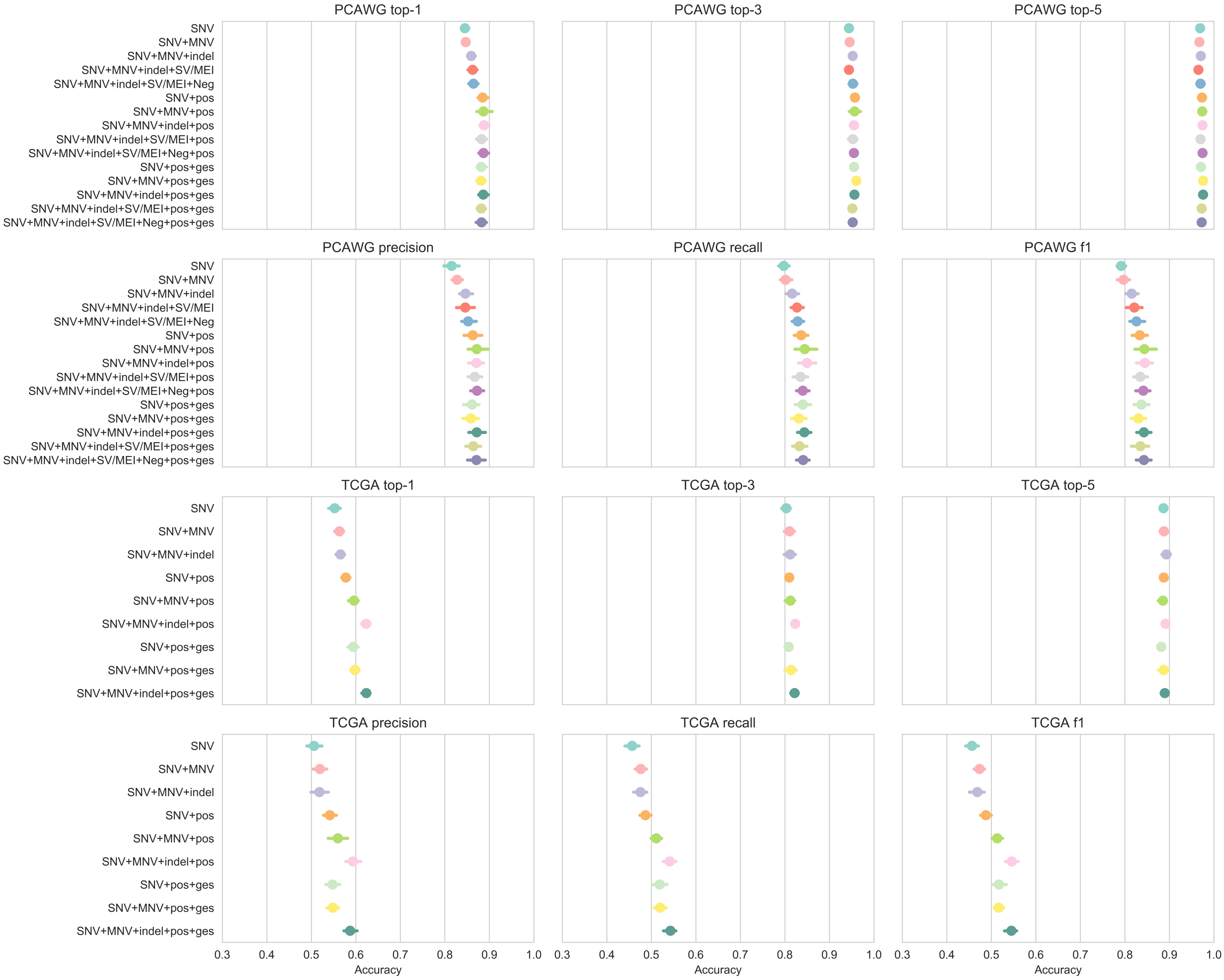

Figure S 2: Top-1, top-3 and top-5 accuracy, precision, recall and F1 scores of MuAt models trained with different mutation types in PCAWG (top) and TCGA (bottom) data. Tests where negative examples were injected into data (see Methods) are denoted with "Neg". Tests where genic, exonic and strand attributes (Methods) were used are denoted with "ges".

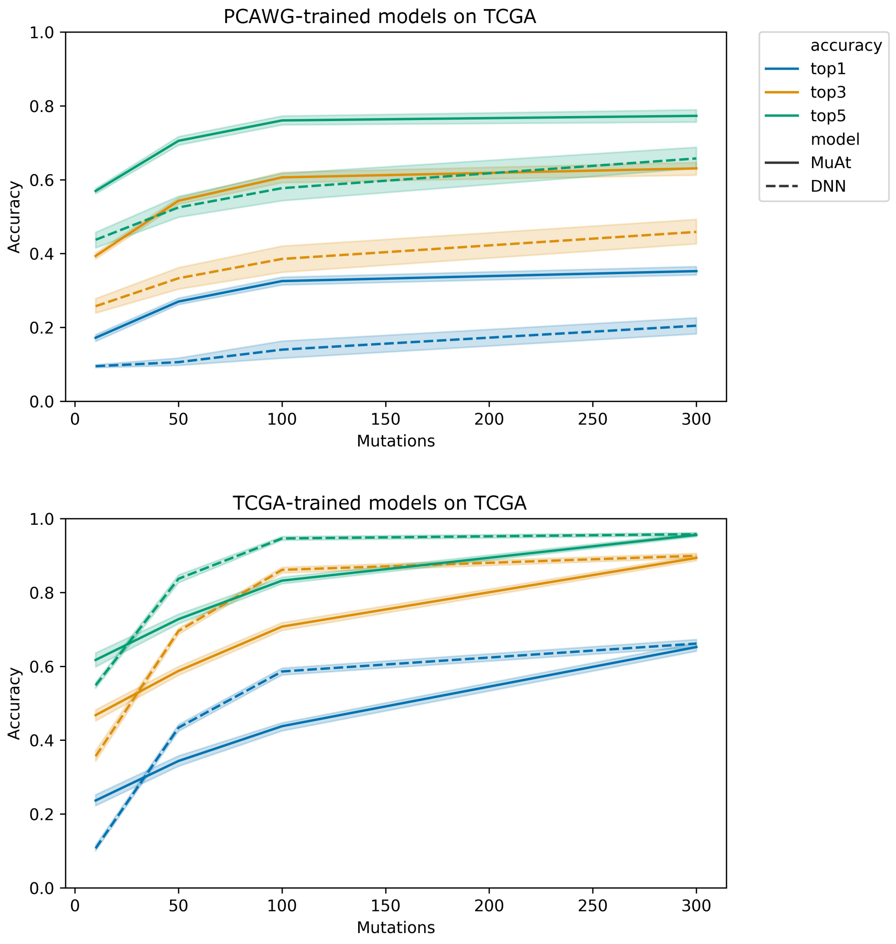

Figure S 3: Transfer learning performance. Top: PCAWG-MuAt on TCGA data. Bottom: TCGA-MuAt on TCGA data. X-axis: maximum number of mutations sampled from each tumour, Y-axis: prediction accuracy.

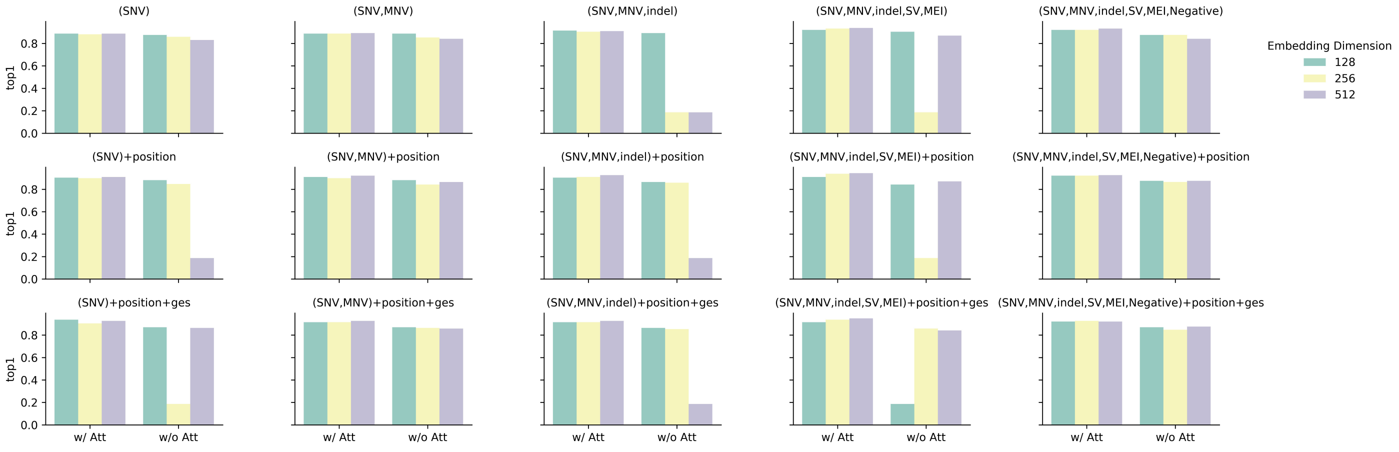

Figure S 4: MuAt model performance with (w/) and without (w/o) the attention module in PCAWG data with different combinations of mutation types.

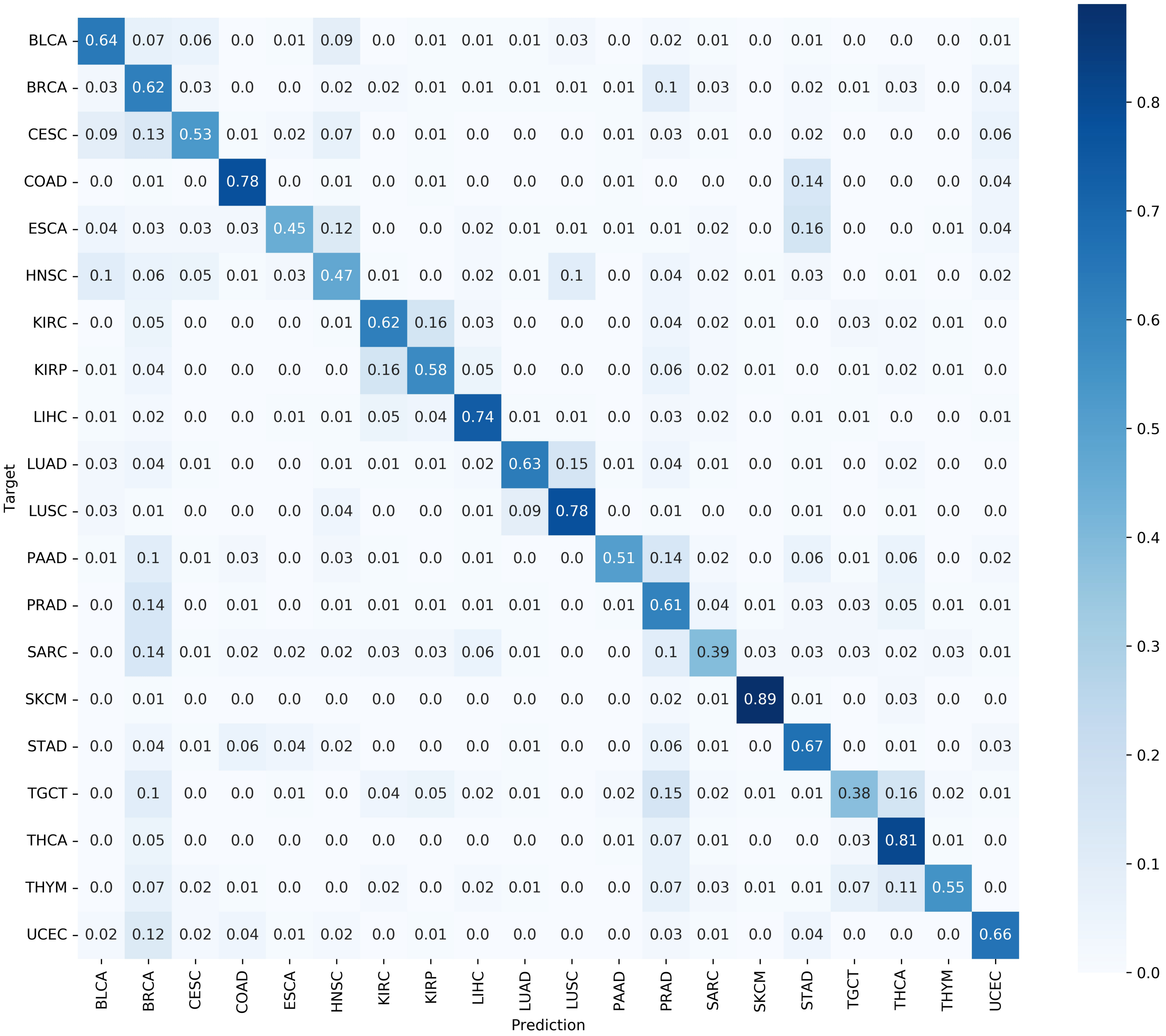

Figure S 5: Confusion matrix of MuAt in TCGA data.

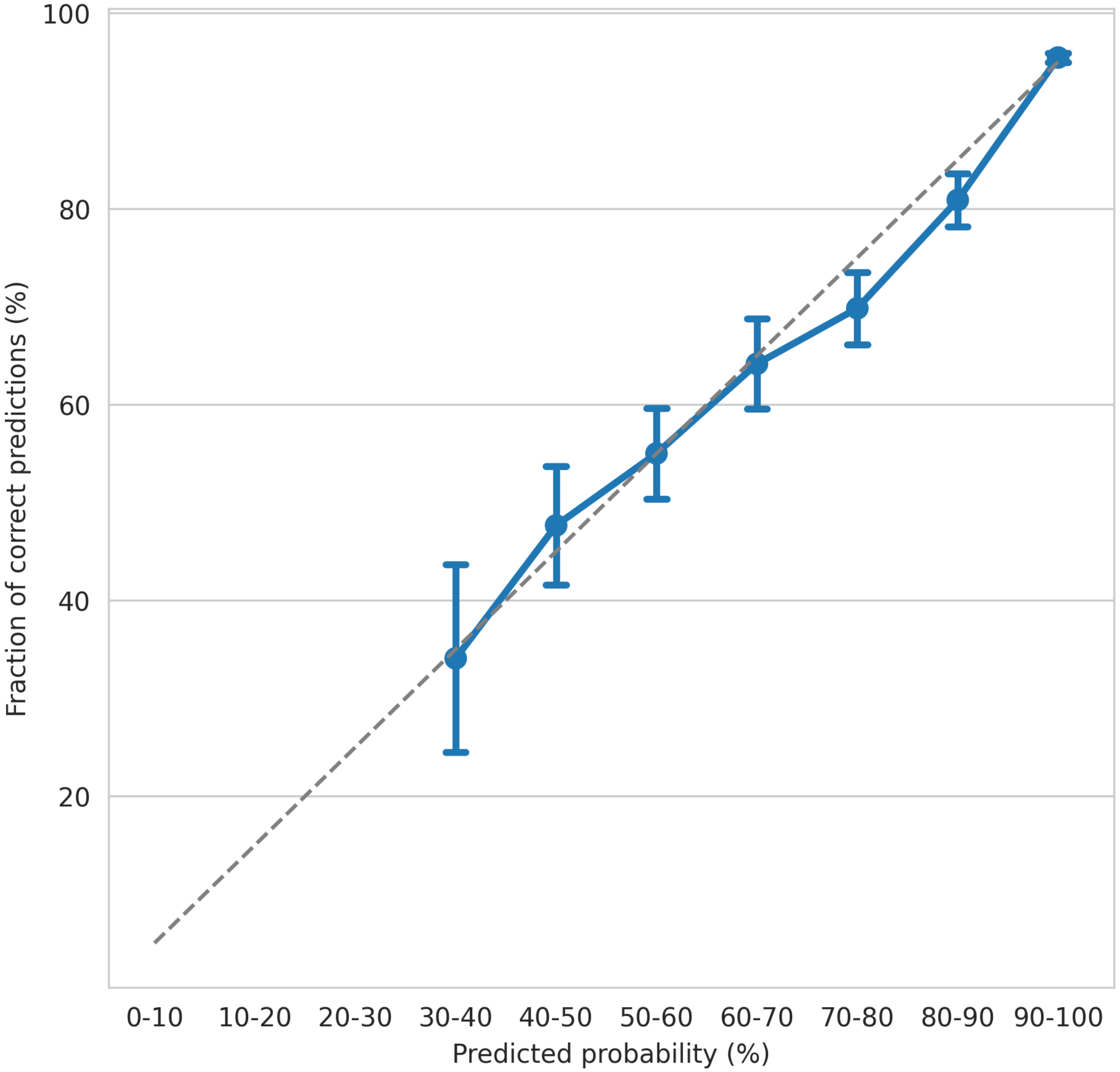

Figure S 6: Calibration curve of MuAt trained with PCAWG data showing the binned predicted probabilities (X-axis) versus the fraction of positive predictions (Y-axis). Error bars indicate standard deviation in bootstrapped data (n=1000). Only bins with more than three tumours shown, excluding the bin (20%,30%) with three tumours.

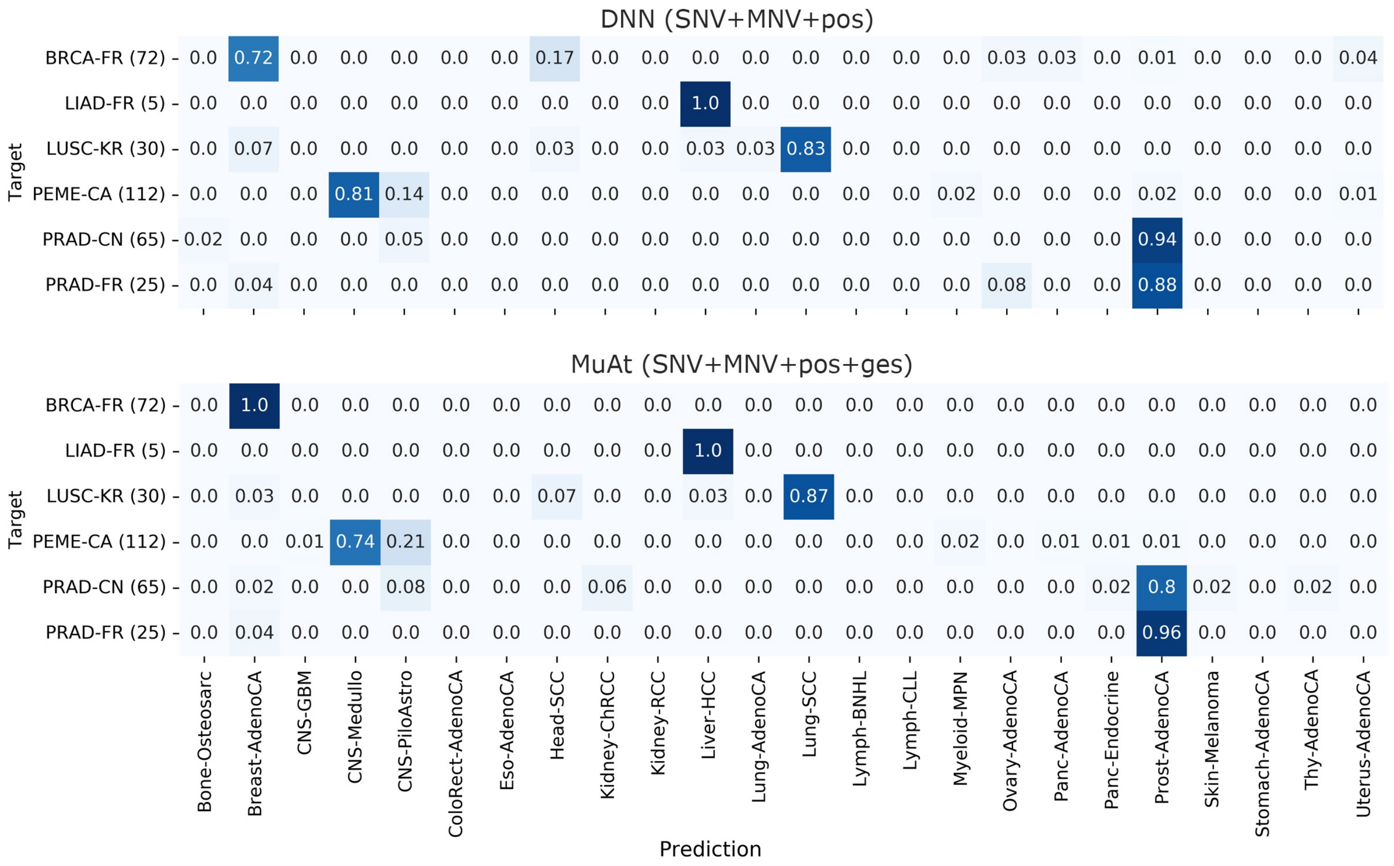

Figure S 7: Confusion matrix for MuAt and DNN ensemble models trained on PCAWG data and evaluated in an independent ICGC whole cancer genome dataset. X-axis: tumour types in PCAWG training data, Y-axis: tumour types of the independent ICGC dataset: Breast cancer (BRCA-FR), Liver hepatocellular carcinoma (LIAD-FR), Lung cancer – Squamous cell carcinoma (LUSC-KR), Pediatric medulloblastoma (PEME-CA) and Prostate cancer (PRAD-CN, PRAD-FR).

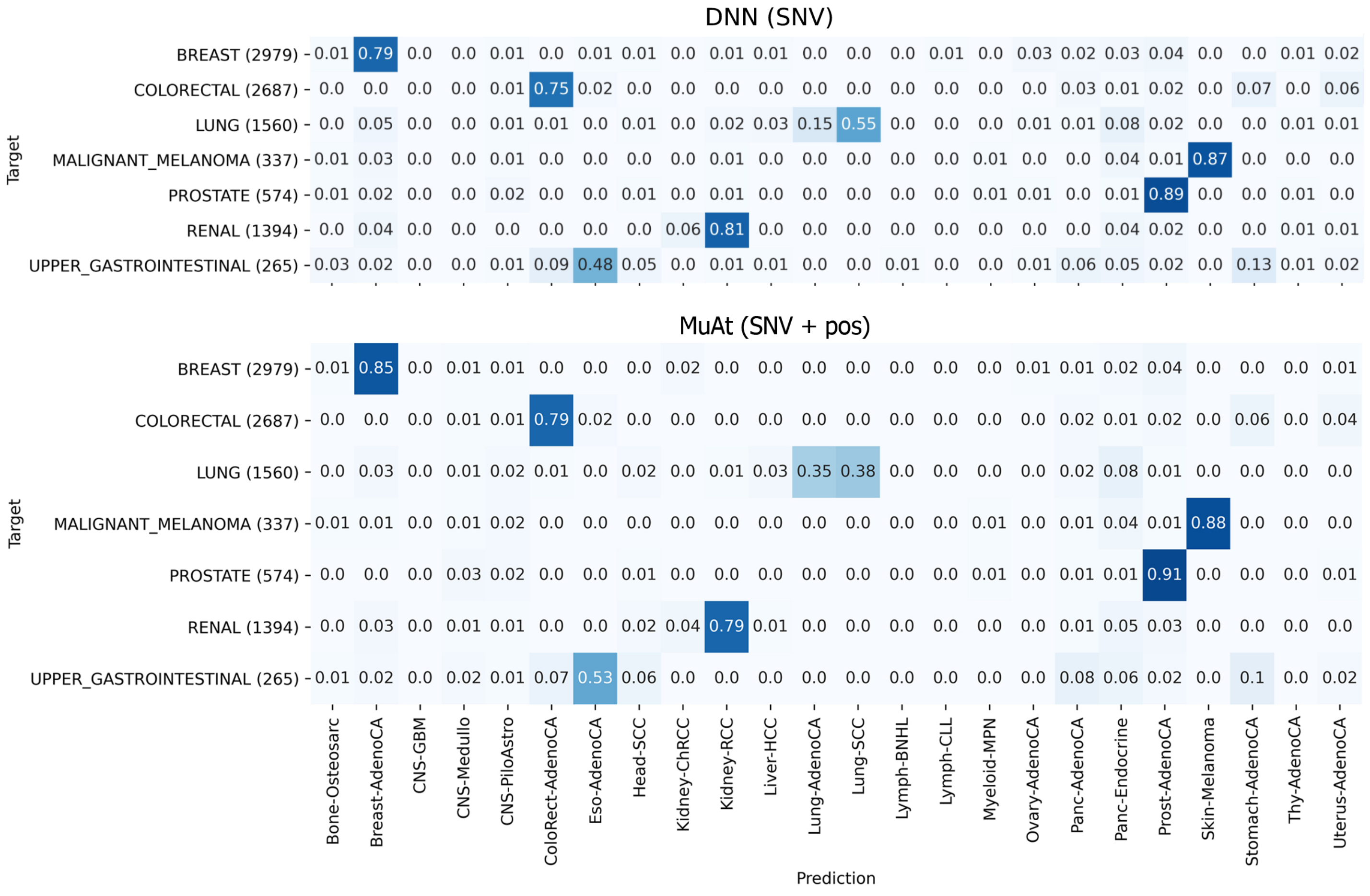

Figure S 8: Confusion matrix for MuAt and DNN ensemble models trained on PCAWG and evaluated on GEL annotated somatic mutations. X-axis: predicted PCAWG tumour types. Y-axis: target GEL tumour types. Results shown for the best ensemble models.

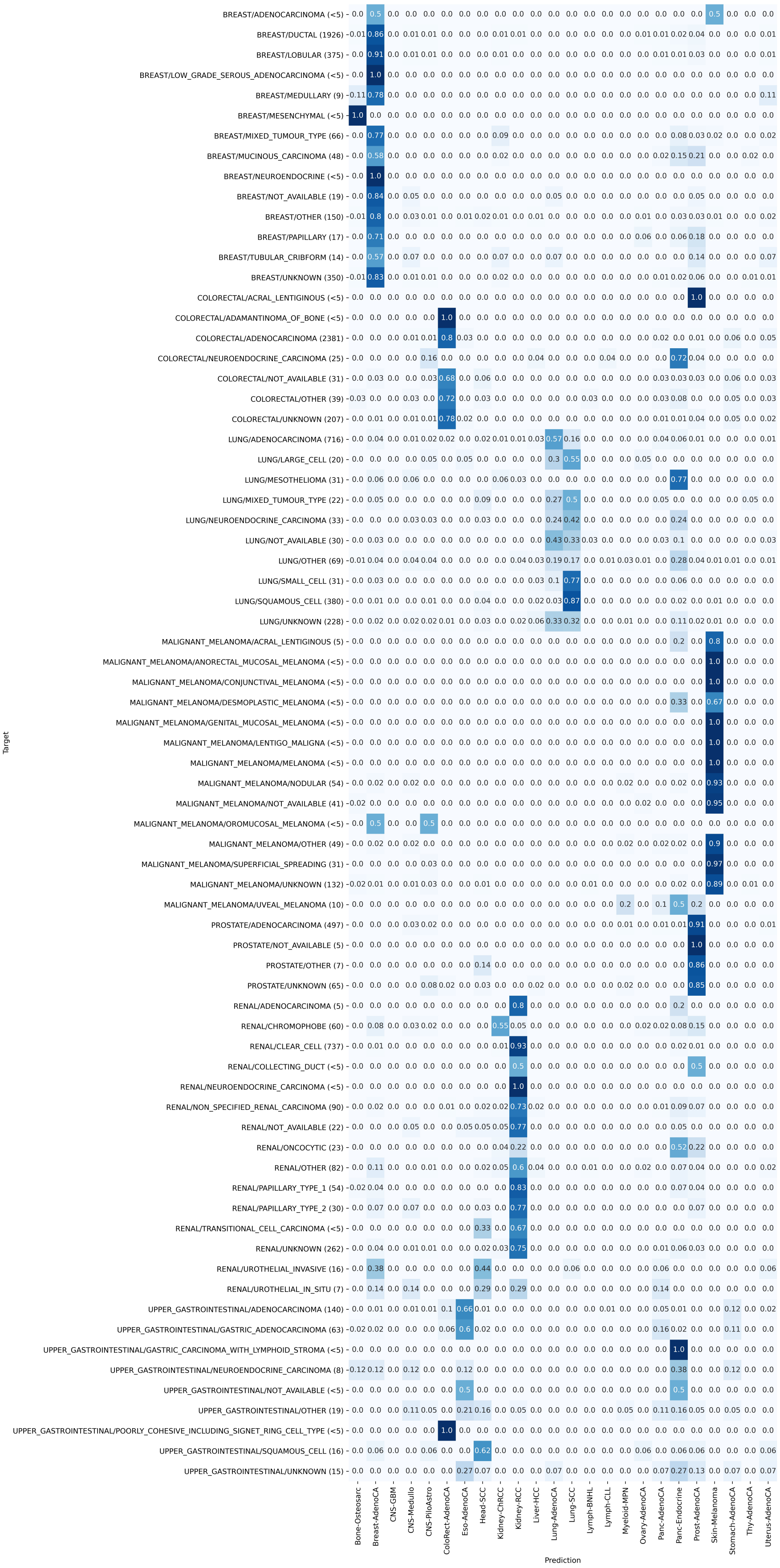

Figure S 9: Confusion matrix for MuAt trained on PCAWG WGS data and evaluated on GEL annotated somatic mutations. X-axis: predicted PCAWG tumour types, Y-axis: target GEL tumour types/subtypes.

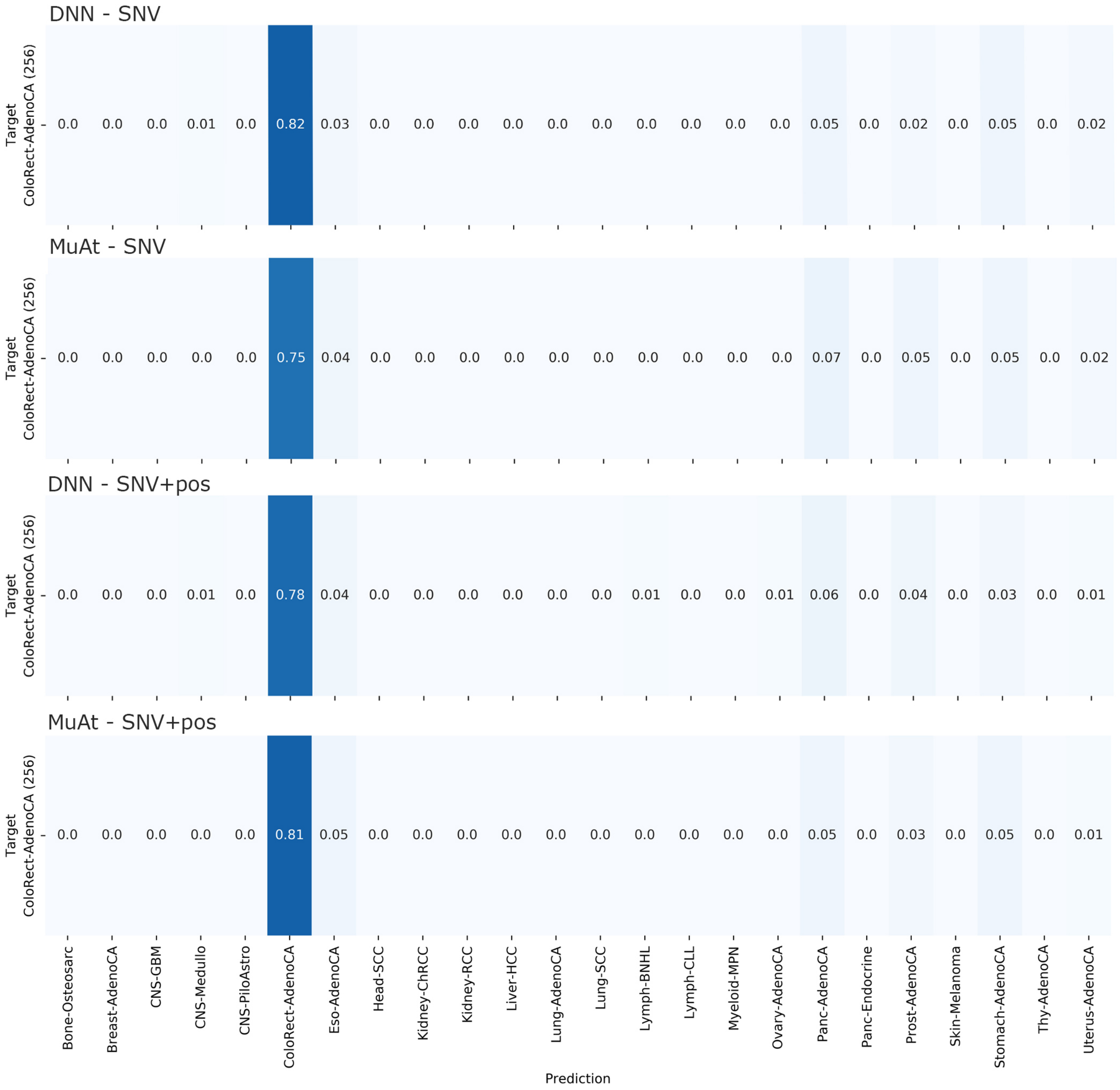

Figure S 10: Confusion matrix for MuAt and DNN ensemble models trained on PCAWG and evaluated on somatic mutations called with MuTect v1.1.4 in whole genomes of 256 CRCs (genome reference GRCh37). X-axis: predicted PCAWG tumour types. Results shown for models trained on SNVs only, and SNVs+genomic positions.

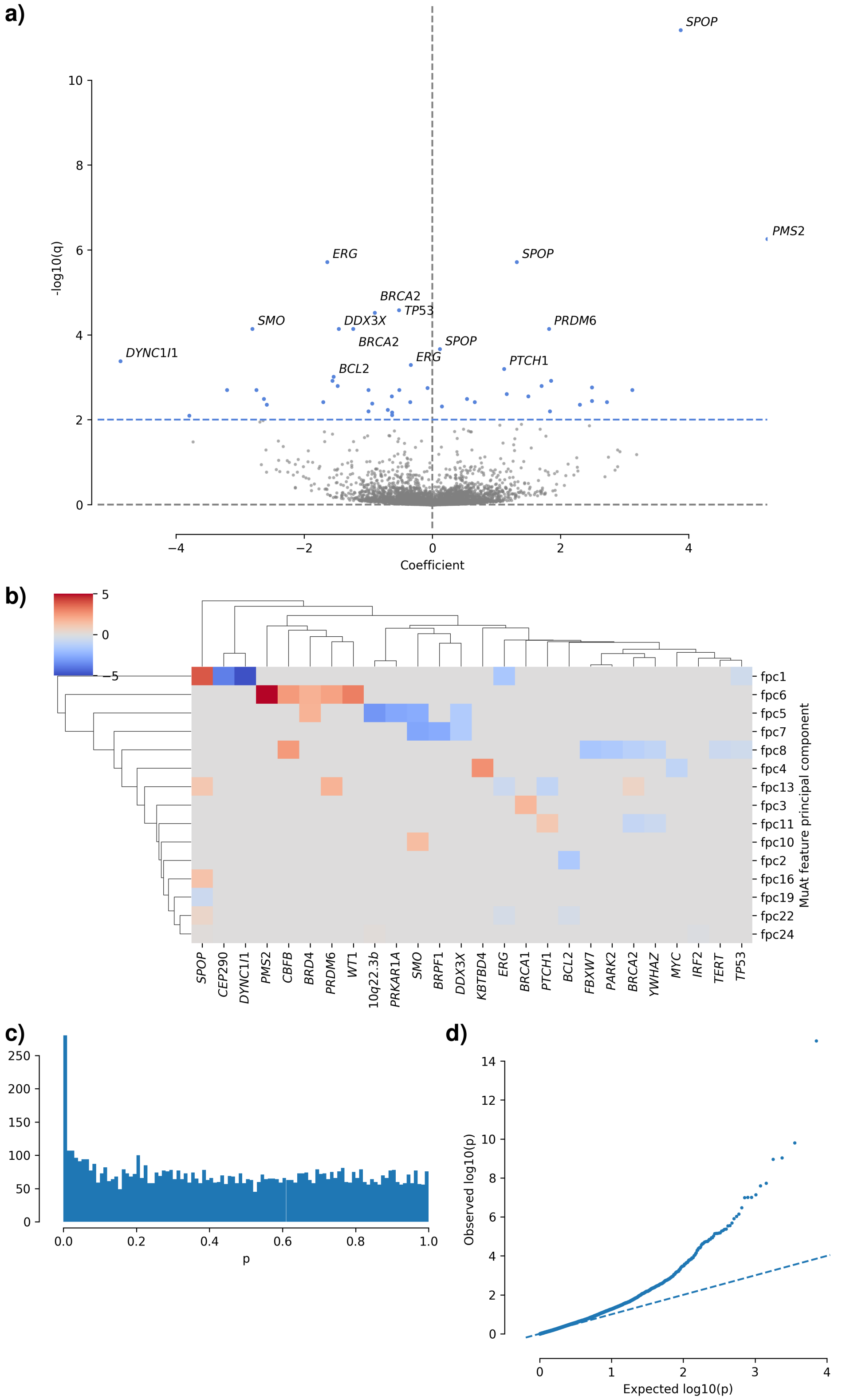

Figure S 11: Linear association of PCAWG driver events with MuAt tumour-level feature principal components (FPCs). Least-squares regression model was corrected with patient age and sex, tumour histology, and first ten principal components of patient genotype. **a)** Volcano plot showing coefficients of all gene-FPC pairs (X-axis) and $\log_{10}(q)$ values (Y-axis). FDR<1% associations are indicated with blue colour, and top 15 associations are labeled. **b)** A clustered heatmap of FDR<1% association coefficients. Enhancer/chr7:86M denotes an enhancer at chr7:86,865,600-86,866,400 (GRCh37). **c)** Histogram and **d)** quantile-quantile plot of p-values.

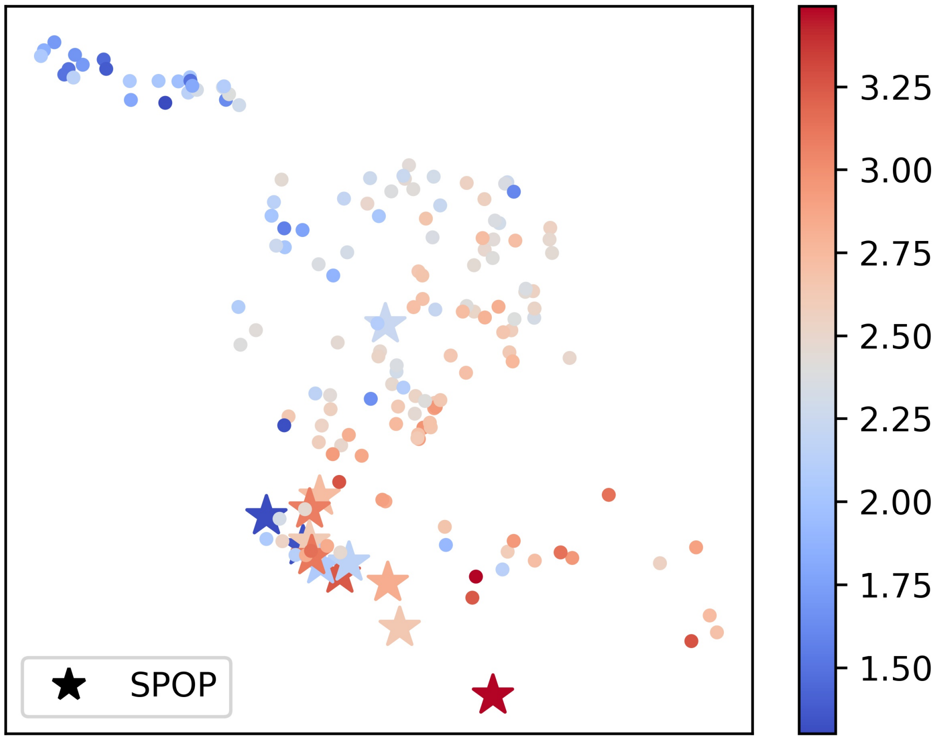

Figure S 12: Prostate cancers with *SPOP* driver events. X- and Y-axis: UMAP components of tumour-level features in the MuAt PCAWG model (coordinates are the same as in Figure 4). Colouring indicates structural variant burden in a log scale.

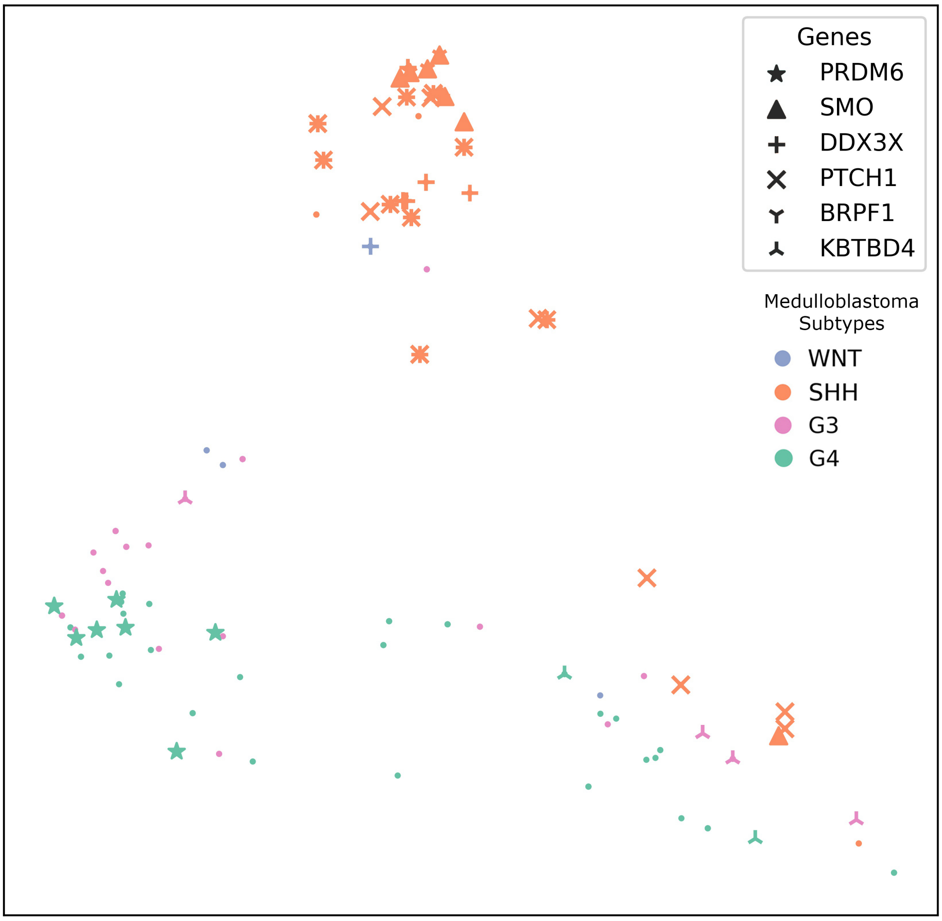

Figure S 13: MuAt tumour-level feature UMAP showing medulloblastomas in the PCAWG dataset (coordinates are the same as in Figure 4). Tumours with driver events associating with MuAt features are highlighted.

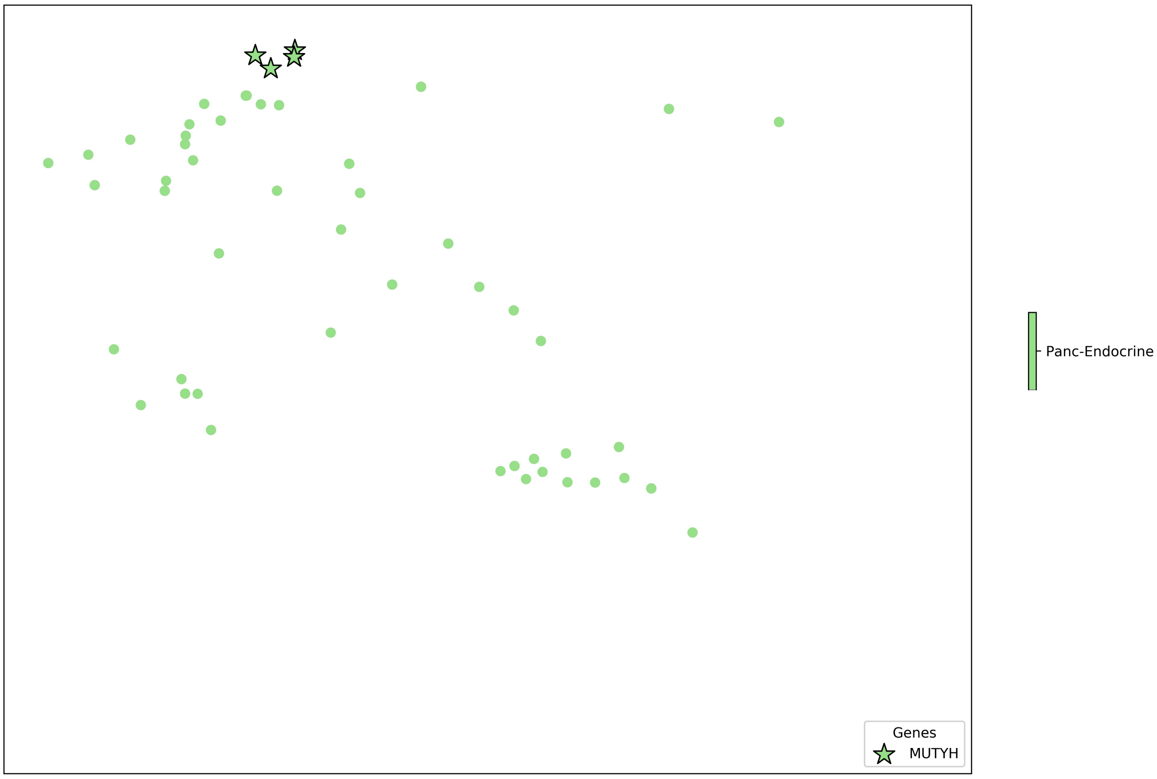

Figure S 14: Pancreatic endocrine tumours of four patients cluster in MuAt tumour-level feature UMAP (coordinates are the same as in Figure 4). These four patients carry germline mutations of *MUTYH*, with loss-of-heterozygosity observed in all four tumours.

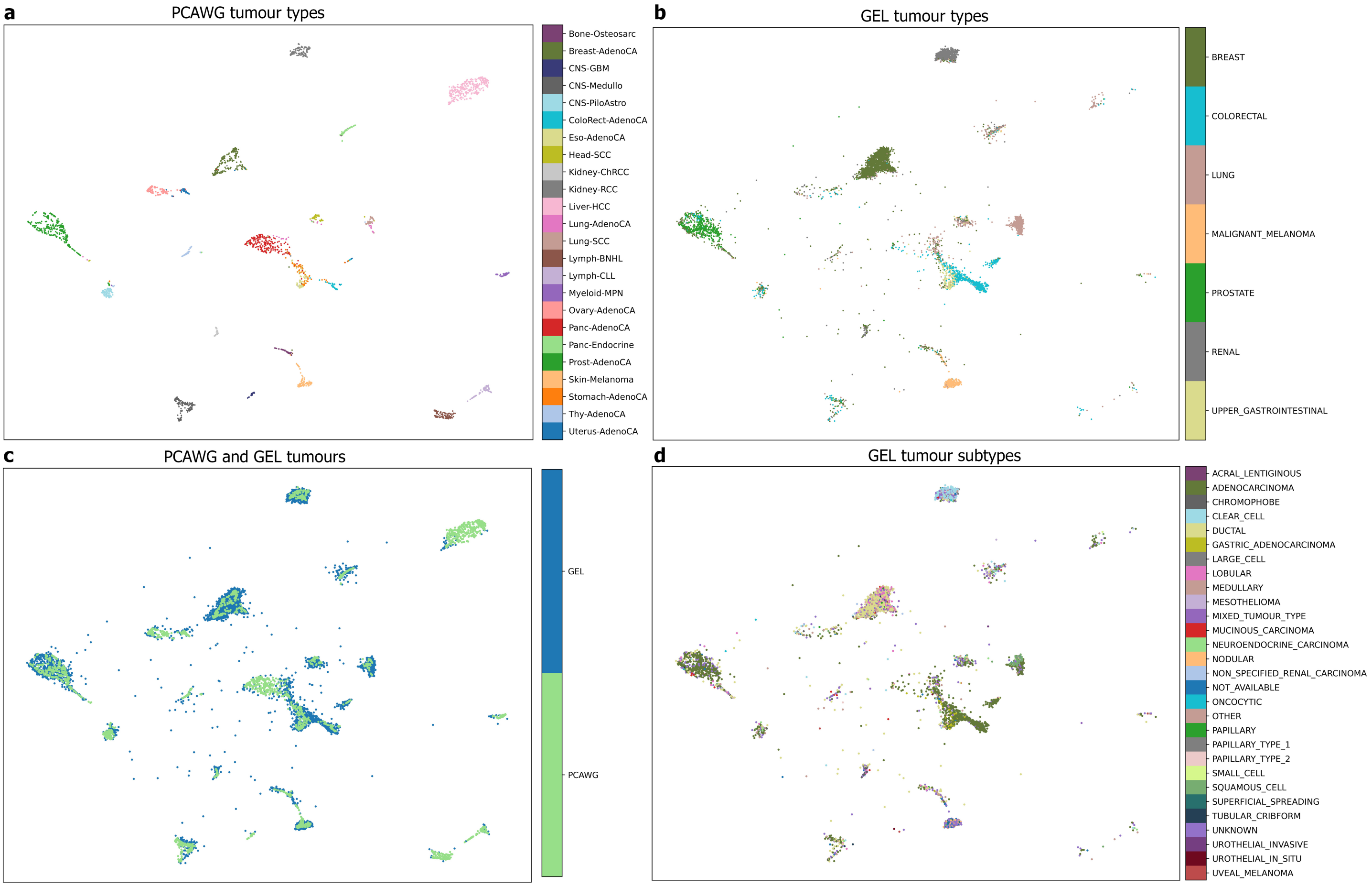

Figure S 15: UMAP of PCAWG and GEL tumour-level features obtained from the MuAt SNV+pos ensemble model trained on PCAWG WGS data. **a**) PCAWG tumours coloured by tumour type. **b**) GEL tumours coloured by tumour type (Cancer Type). **c**) PCAWG and GEL tumours. **d**) GEL tumours coloured by tumour subtype (Cancer Sub Type).

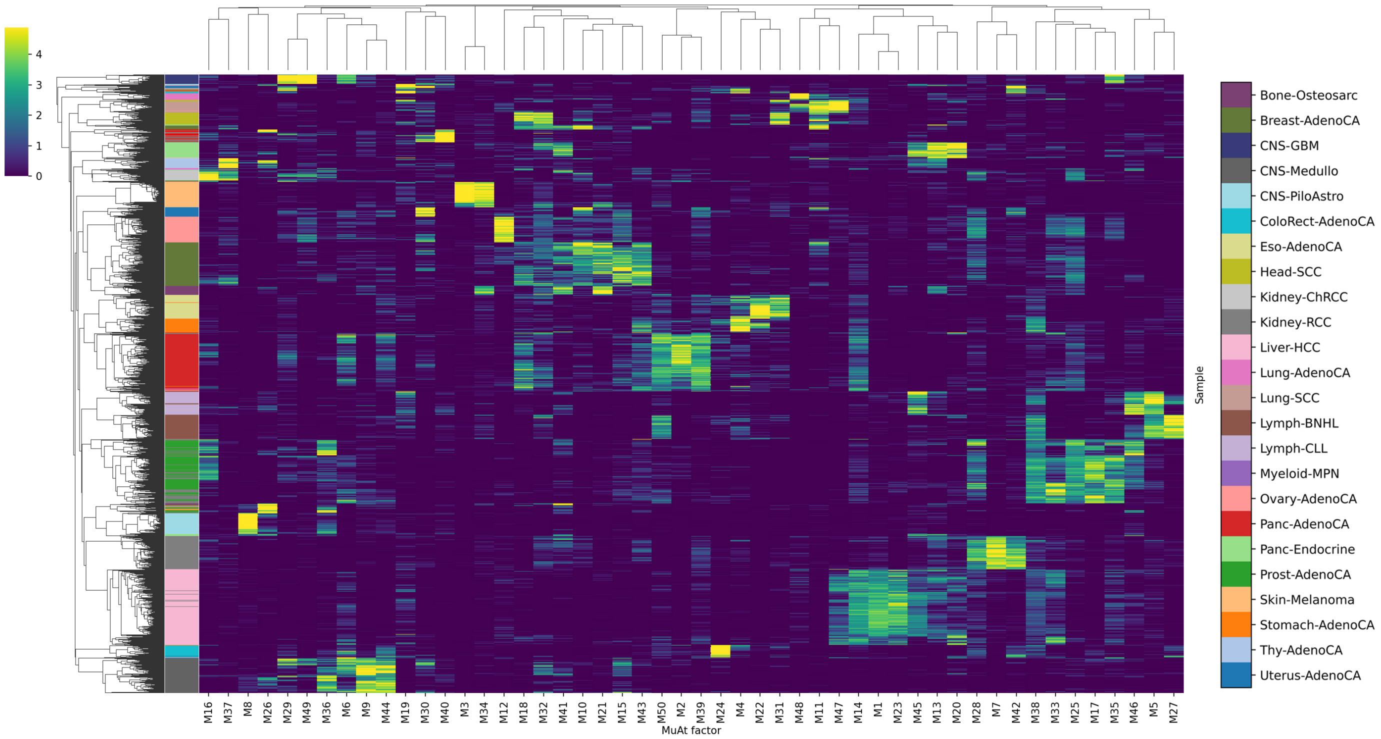

Figure S 16: A clustered heatmap of MuAt tumour-level factors in each PCAWG tumours.

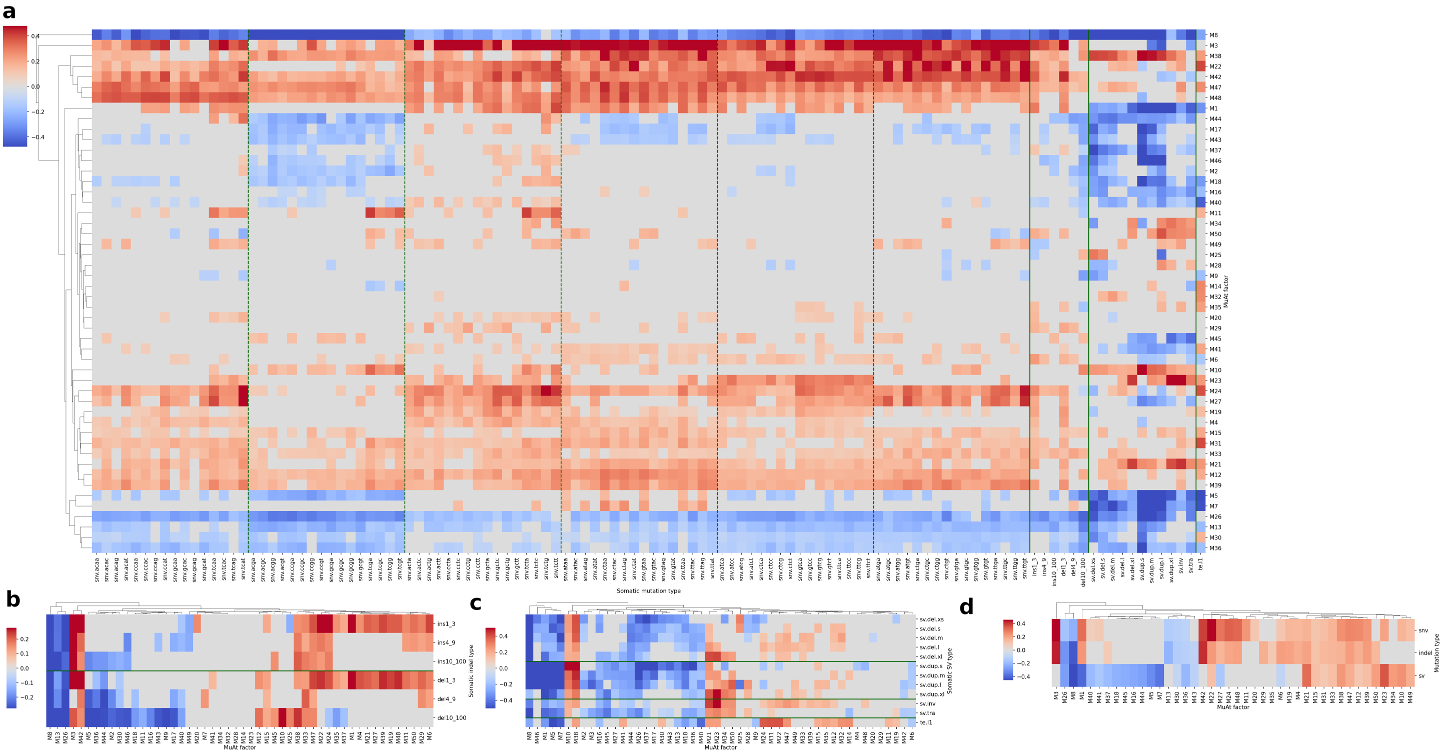

Figure S 17: Association of MuAt tumour-level factors (Y-axis) with mutation counts by type (X-axis). A negative binomial model was used to predict counts of each mutation type given MuAt factors M1,. . .,M50 in PCAWG data. FDR<5% results shown as non-grey colours. **a**) All somatic mutation types. **b**) indels only. **c**) SVs only. **d**) association to mutation type counts. Event size of SV deletions and duplications is denoted by xs (<1 kb), s (1-10 kb), m (10-100 kb), l (100-1000 kb) and xl (>1000 kb).

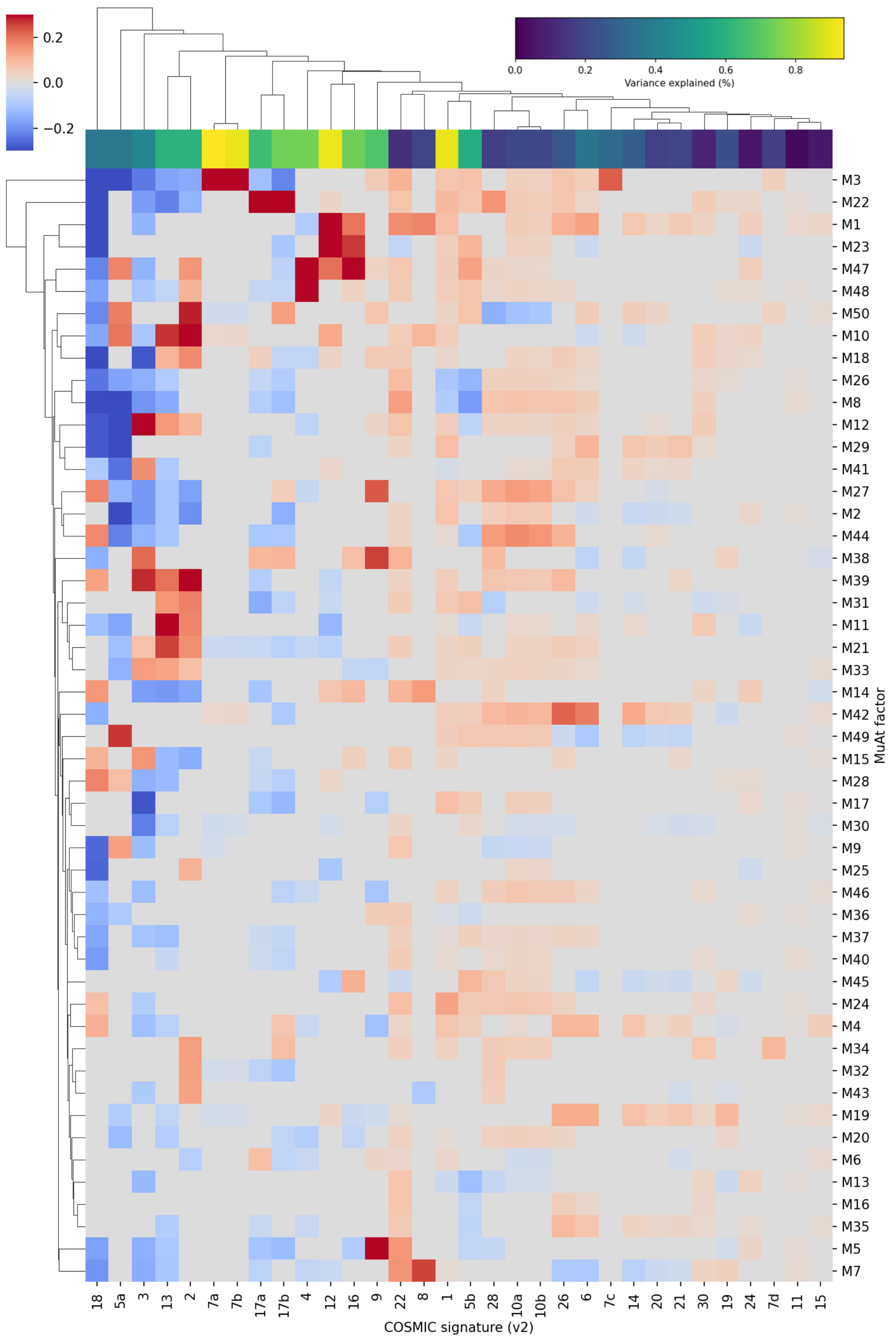

Figure S 18: Association of MuAt PCAWG model tumour-level factors (Y-axis) with COSMIC mutational signatures (version 2; X-axis). Log-transformed number of mutations attributed to each signature was predicted with least-squares regression given MuAt factors. FDR<10% associations shown in non-grey colours. Variance of each signature explained indicated on the top row.

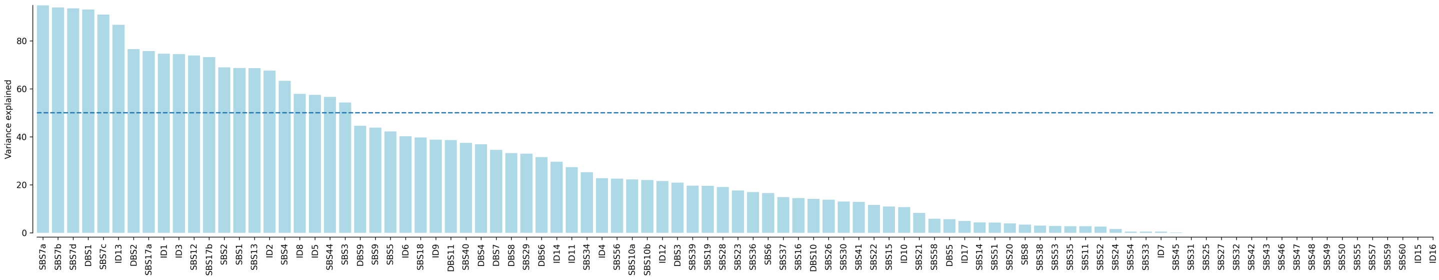

Figure S 19: Variance in the number of mutations attributed to signatures (COSMIC version 3) explained by MuAt tumour-level factors.

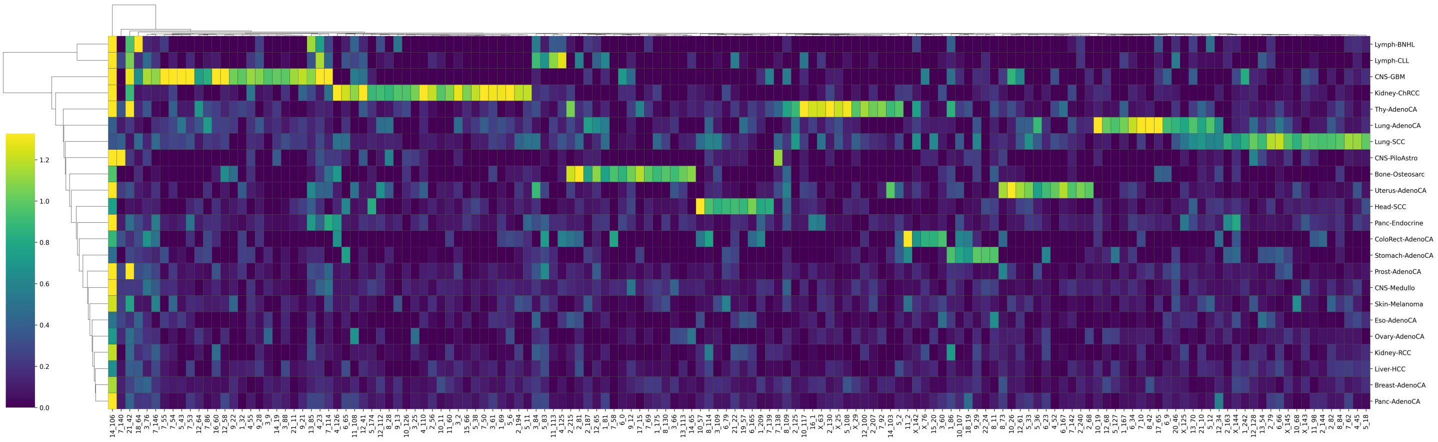

Figure S 20: Mean attention values of genomic positions (X-axis) per tumour type (Y-axis) in the MuAt PCAWG model. Genomic positions given as "chromosome_b", where b is the 1-Mbp bin starting at chromosome position $b \times 10^6$. Genomic positions with the highest 5% variability in attention across tumours are shown. Many genomic position bins appear characteristic to specific tumour types. Genomic region chr14:106,000,000-107,000,000 contains the *IGH* region (leftmost bin), which is recurrently mutated in B cells.
