# Supplementary material for: Mutation-Attention (MuAt): deep representation learning of somatic mutations for tumour typing and subtyping
Source: Genome Med. 2023 Jul 7;15:47. doi: 10.1186/s13073-023-01204-4 (PMC10326961; doi:10.1186/s13073-023-01204-4)
Supplement: Supplementary file 3 — Additional file 3. The Genomics England Research Consortium. [file 13073_2023_1204_MOESM3_ESM.pdf]

Genomics England Research Consortium

The Genomics England Research Consortium

Ambrose, J. C. [1]; Arumugam, P.[1]; Bevers, R.[1]; Bleda, M. [1]; Boardman-Pretty, F. [1,2]; Boustred, C. R. [1]; Brittain, H.[1]; Brown, M.A.; Caulfield, M. J.[1,2]; Chan, G. C. [1]; Giess A. [1]; Griffin, J. N.; Hamblin, A.[1]; Henderson, S.[1,2]; Hubbard, T. J. P. [1]; Jackson, R. [1]; Jones, L. J. [1,2]; Kasperaviciute, D. [1,2]; Kayikci, M. [1]; Kousathanas, A. [1]; Lahnstein, L. [1]; Lakey, A.; Leigh, S. E. A. [1]; Leong, I. U. S. [1]; Lopez, F. J. [1]; Maleady-Crowe, F. [1]; McEntagart, M.[1]; Minneci F. [1]; Mitchell, J. [1]; Moutsianas, L. [1,2]; Mueller, M. [1,2]; Murugaesu, N. [1]; Need, A. C. [1,2]; O'Donovan P. [1]; Odhams, C. A. [1]; Patch, C. [1,2]; Perez-Gil, D. [1]; Pereira, M. B.[1]; Pullinger, J. [1]; Rahim, T. [1]; Rendon, A. [1]; Rogers, T. [1]; Savage, K. [1]; Sawant, K. [1]; Scott, R. H. [1]; Siddiq, A. [1]; Sieghart, A. [1]; Smith, S. C. [1]; Sosinsky, A. [1,2]; Stuckey, A. [1]; Tanguy M. [1]; Taylor Tavares, A. L.[1]; Thomas, E. R. A. [1,2]; Thompson, S. R. [1]; Tucci, A. [1,2]; Welland, M. J. [1]; Williams, E. [1]; Witkowska, K. [1,2]; Wood, S. M. [1,2]; Zarowiecki, M. [1].

1. Genomics England, London, UK

2. William Harvey Research Institute, Queen Mary University of London, London, EC1M 6BQ, UK.

8th May 2022
